# Discovery of *Gibellula floridensis* from Infected Spiders and Analysis of the Surrounding Fungal Entomopathogen Community

**DOI:** 10.3390/jof10100694

**Published:** 2024-10-04

**Authors:** Ross A. Joseph, Abolfazl Masoudi, Mateo J. Valdiviezo, Nemat O. Keyhani

**Affiliations:** 1 Department of Biological Sciences, University of Illinois at Chicago, Chicago, IL 60607, USA; rossj@uic.edu (R.A.J.); amasou7@uic.edu (A.M.); 2 Department of Microbiology and Cell Science, University of Florida, Gainesville, FL 32611, USA; mvaldiviezo@ufl.edu

**Keywords:** spider, insect and spider fungal pathogens, *Gibellula*, habitat, amplicon sequencing

## Abstract

Characterization of fungal spider pathogens lags far behind their insect counterparts. In addition, little to nothing is known concerning the ecological reservoir and/or fungal entomopathogen community surrounding infection sites. Five infected spider cadavers were identified in the neo-tropical climate of north-central Florida, USA, from three of which viable cultures were obtained. Multi-locus molecular phylogenetic and morphological characterization identified one isolate as a new *Gibellula* species, here named, *Gibellula floridensis*, and the other isolates highly similar to *Parengyodontium album*. The fungal entomopathogen community surrounding infected spiders was sampled at different habitats/trophic levels, including soil, leaf litter, leaf, and twig, and analyzed using ITS amplicon sequencing. These data revealed broad but differential distribution of insect-pathogenic fungi between habitats and variation between sites, with members of genera belonging to *Metarhizium* and *Metacordyceps* from Clavicipitaceae, *Purpureocillium* and *Polycephalomyces* from Ophiocordyceps, and *Akanthomyces* and *Simplicillium* from Cordycipitaceae predominating. However, no sequences corresponding to *Gibellula* or *Parengyodontium*, even at the genera levels, could be detected. Potential explanations for these findings are discussed. These data highlight novel discovery of fungal spider pathogens and open the broader question regarding the environmental distribution and ecological niches of such host-specific pathogens.

## 1. Introduction

Spiders (Arachnida: Araneae) are highly diverse predatory arthropods characteristic for their use of silk and venom to capture and immobilize prey [1,2,3]. Occupying a wide range of ecosystems, these organisms play critical ecological roles by feeding on species of insects, fish, reptiles, and even birds and mammals [4,5,6]. Similar to insects, a major driver of spider mortality appears to be infection by entomopathogenic fungi, including species in the Cordycipitaceae, Ophiocordycipitaceae, and Clavicipitaceae families [7,8,9,10,11]. Among spider-infecting fungi, *Gibellula* (Cordycipitaceae) is a genus of specialized obligate spider-infecting pathogens with ~31 species described thus far, mainly from tropical and temperate environments [12,13,14,15,16,17,18]. These fungi are often characterized by causing their spider hosts to attach themselves to the undersides of leaves during the infection process, where the fungus kills it, covering the cadaver in a thick layer of mycelia and sprouting synnemata which produce conidia that are released to “rain” down on new unsuspecting victims [19]. Thus far, *Gibellula* species have been described to infect over 15 different spider families, although identifying spider hosts can be challenging as the fungus envelops the spider body, obscuring morphological features of the infected organism [13]. Despite the apparent global distribution of this genus, knowledge concerning the diversity of *Gibellula* species in many geographic regions are lacking, with most described species isolated from tropical regions of either Southeast Asia or South America [13,14,15,20,21,22,23]. Indeed, to date, only two *Gibellula* species have been reported from North America, *G. leiopus* and *G. pulchra*, primarily from infected spiders of the Trachelidae and Salticidae families [13,24].

Aside from a lack of knowledge concerning *Gibellula* species diversity in many areas of the world, almost nothing is known concerning their environmental prevalence and/or the fungal community dynamics surrounding infection sites. Some genera of entomopathogenic fungi, particularly generalist facultative fungi of the Cordycipitaceae and Clavicipitaceae families, such as *Beauveria* and *Metarhizium* sp., which have broad host ranges, readily grow in vitro on mycological media and can be found outside of their insect hosts in environmental reservoirs such as soil and/or can be associated in the plant rhizosphere or even as plant epi-/endophytes [25,26,27,28]. In contrast, specialized insect pathogenic fungi of the order Entomophthorales, e.g., *Massospora cicadina*, *Arthrophaga myriapodina*, and *Entomophthora muscae*, are obligate insect pathogens and are not readily culturable (if at all) in vitro [29,30,31]. Some of these latter fungi, e.g., *Massospora* sp., produce resting spores that can persist in the soil [32,33,34]. *Gibellula* sp. present an in between phenotype, where they can be cultured in vitro, i.e., can grow saprophytically, but have narrow, specialized host ranges. Furthermore, though poorly examined, *Gibellula* infection apparently includes some level of manipulation of host behavior, as part of its extended phenotype is to cause their hosts to become fixed to the underside of leaves and other plant material, often at an elevated position above the ground [35,36]. This presumably facilitates the spread spores onto the surrounding area in order to infect other hosts. However, to our knowledge, to date there are no reports attempting to detect *Gibellula* outside of their spider hosts and/or to examine the fungal community surrounding infected hosts. Such studies are important as little is known concerning any potential reservoir for host-specific insect fungal pathogens and/or the fungal community dynamics that may affect infection, persistence, and/or transmission/spread of the fungus.

In the present study, we found the cadavers of five fungal infected spiders in two forested locations surrounding the University of Florida in Gainesville, Florida: Lake Alice Natural Area and Loblolly Woods Nature Park. Based on visible morphological characteristics, all spider hosts appeared to be of a single genus, *Trachelas*, and all were found to have fixed their bodies in similar positions and on similar substrates: to bark on the undersides of tree branches and trunks one to two meters above the ground. These specimens were collected, and cultures were obtained from three of the five cadavers. Through phylogenetic characterization of cultured isolates using ITS, LSU, SSU, and TEF1a loci as well as morphological characterization, one isolate was confirmed as a new *Gibellula* species, herein described as *Gibellula floridensis* and the other two isolates as *Parengyodontium album* (Cordycipitaceae). The morphology of these isolates was characterized on different growth media, and the characters of the *Gibellula* isolate are described. *Gibellula floridensis* is most closely related to *G. leiopus* but differs by the appearance of their synnemata, as well as the length of their conidiophores, metulae, and conidia. To determine the presence and prevalence of these isolates outside of their spider host, a systematic survey of the fungal communities of samples derived from four trophic levels, namely, soil, leaf litter, leaves, and twigs, from areas surrounding the location of the spider cadavers was performed via amplicon sequencing of the ITS1 region. These areas were found to contain high abundances and diversity of entomopathogenic fungi, particularly in soil samples, with differential distribution between the trophic levels examined. However, sequences corresponding to the *Gibellula* or *Parengyodontium* species isolated in our survey were not detected in these samples, nor, surprisingly, were sequences corresponding to other *Gibellula* or *Parengyodontium* species observed in our dataset. Potential explanations for failure to detect the characterized isolates are discussed. This study adds to the known diversity of *Gibellula* species in North America and opens questions of the presence, prevalence, and distribution of *Gibellula* and other entomopathogenic fungi outside of their arthropod hosts.

## 2. Materials and Methods

### 2.1. Infected Spider Location and Collection

Spiders infected by fungal pathogens were located and collected from the Lake Alice and Loblolly natural areas in Gainesville, Florida, United States over the course of several months between March and August 2023. During each collection, the trunks, branches, twigs, and leaves of trees were carefully examined for the presence of spider cadavers. Once located, cadavers were photographed in situ prior to removal. Cadavers were collected from locations on tree trunks and branches using a knife to remove a small amount of the bark to which the spiders had become firmly attached. Collected samples were stored in Falcon tubes or sealed Petri dishes until they could be returned to the lab for further investigation. Once in the lab, samples were stored in a cool dark environment for further photographic documentation and culturing of the infecting fungi.

### 2.2. Culturing from Infected Spiders and Assessing Growth on Different Media Types

Pure cultures were obtained from infected spider cadavers by lightly touching the conidiophores forming at the ends of synnemata growing from the spiders with a sterilized inoculation loop and then streaking this loop in quadrants onto a potato dextrose agar (PDA) plate containing 200 μg/mL of streptomycin and ampicillin. The plates were monitored for the presence of fungal colonies, and, once appearing, these colonies were touched with an inoculation loop and spread onto a new plate as above for single-spore isolation of cultures. Single-spore isolation was repeated three times for each culture to obtain pure cultures, characterized by morphologically uniform growth. This technique produced pure cultures of a single fungal isolate from spiders 3, 4, and 5, with the isolates being designated UFSI_3, UFSI_4, and UFSI_5, respectively. Once pure cultures of each isolate were obtained, plates containing PDA, Sabouraud dextrose agar (SDA), malt extract agar (MEA), and Czapek–Dox solution agar (CZA) were prepared, and 1 mm plugs of all isolates were taken from the edges of actively growing cultures and inoculated into the center of these plates. Colony growth and morphology were assessed over the course of 1 month for each isolate on each of the above media types to determine growth rates and substrate preferences.

### 2.3. Environmental Sampling

Soil samples were collected at the locations shown (Supplementary Figure S1), and the soil-sampling sites were assigned coordinates via the Global Positioning System (GPS). The two areas sampled where infected spiders were found were (1) Lake Alice (LA) (29°38′31.14″ N, 82°21′29.20″ W) and (2) Loblolly Woods Nature Park (LB) (29°39′34.74″ N, 82°22′7.27″ W) both in Gainesville, Florida. The LA region was treated as a single site, from which 3 soil, 3 plant litter, 2 twig, and 2 leaf samples were collected. The LB region was divided into four sites, from which 3 soil and 3 plant litter samples were collected from each site, along with 1 twig and 1 leaf sample per site. From the LB region, a total of 12 soil samples, 12 plant litter samples, 4 twig samples, and 4 leaf samples were collected. In total, 42 samples were collected from both regions. Sample collection followed a standard protocol [26]. Briefly, at each location, soil sampling involved marking out five transects spaced ~100 m apart. Along each line, the soil was sampled at five points, each 10 m apart, starting 50 m from the beginning of the line. Samples were taken from the topsoil down to a depth of 30–50 cm and had a diameter of 30 cm. These subsamples were collected using clean shovels and combined in a plastic bag to create a single mixed sample per line. Efforts were made to remove large roots and gravel bigger than 0.5 cm from the samples. The shovels were rinsed with 70% alcohol and dried before moving to the next location. Subsamples of the mixed soil were then packed into individually labeled zip-lock bags (10 cm × 12 cm), and for each site, five of these bagged samples were stored in a large, sealable polyethylene bag and kept cool in a cooler. The schematic for the soil sampling is presented in Supplementary Figure S1.

For isolation of the microbial communities, samples from the plant litter, leaves, and twigs were entirely submerged in 500 mL of distilled water and agitated for three hours at 140 revolutions per minute (rpm) to solubilize the microbial communities from the environmental sample surfaces into the water. Suspensions were filtered through a 0.22 μm pore-size mixed-cellulose esters (MCEs) membrane filter (MF-Millipore^TM^, Tullagreen, Carrigtwohill, Ireland) to collect the microbial community. Filters were immediately frozen and stored at −80 °C.

### 2.4. DNA Extraction, PCR, and Sequencing

To extract DNA from cultured isolates, a small plug of actively growing hyphae at the edge of a culture plate was removed and placed into a flask containing 50 mL of potato dextrose broth (PDB) to initiate liquid cultures. Liquid cultures were grown at 25 °C with 250 rpm orbital shaking for 7 d, at which point small balls of hyphae could be noted at the bottom of the culture medium. Hyphae were separated from the culture medium by filtration through a 0.7 μm filter and washed with sterile distilled water to remove any residual culture media. Filtered hyphae were then collected into 2 mL tubes along with a sterile glass bead and lyophilized overnight until all water had been removed from the tissues. Lyophilized tissue was powdered by bead beating using an MP FastPrep-24 (MP Biomedicals, Irvine, CA, USA) at 4.0 m/s for 60 s. DNA was extracted from powdered tissues using the Norgen Biotek Plant/Fungi DNA Isolation Kit (Norgen Biotek, Thorold, ON, Canada) according to manufacturer’s instructions. Concentration and purity of extracted DNA was assessed using an NP80 nanophotometer (Implemen, Munich, Germany). To amplify loci for downstream sequencing and phylogenetic analysis, the primer sets ITS1F (5′-CTTGGTCATTTAGAGGAAGTAA-3′)/ITS4 (5′-TCCTCCGCTTATTGATATGC-3′) (ITS region), LROR-F (5′-ACCCGCTGAACTTAAGC-3′)/LR5-R (5′-TCCTGA GGGAAACTTCG-3′) (LSU region), NS1-F (5′-GTAGTCATATGCTTGTCTC-3′)/NS4-R (5′-CTTCCGTCAATTCCTTTAAG-3′) (SSU region), and EF1-728F (5′-CATCGAGAAGTTCGAGAAGG-3′)/TEF1LLErev (5′-AACTTGCAGGCAATGTGG-3′) (TEF1a region) were used in conjunction with taq 5x master mix (New England Biosciences, Ipswich, MA, USA) according to manufacturer instructions. The success of all polymerase chain reactions (PCRs) were verified by gel electrophoresis to visually confirm the presence of a single amplified band. PCR products were purified using GeneJET PCR Purification Kit (Thermo Scientific, Waltham, MA, USA) according to manufacturer instructions, and the concentration was verified by nanophotometer as above prior to sending samples for sequencing. PCR products were sequenced using Oxford Nanopore-sequencing technology (Plasmidsaurus, Eugene, OR, USA). The consensus sequences received from the nanopore-sequencing results were assessed by BLAST searching and examining CDS features to determine sequence quality, strandedness, and the presence of introns. Sequences for each isolate were deposited in Genbank under the following accession numbers: UFSI_3: PP915745 (ITS), PP915999 (LSU), PP916007 (SSU); UFSI_4: PP915746 (ITS), PP916000 (LSU), PP916008 (SSU); UFSI_5: PP915747 (ITS), PP916001 (LSU), PP916009 (SSU), PP938454 (TEF1a).

For environmental samples (soil, leaves, leaf litter, and twigs), total DNA from soil and those from samples collected on 0.22 μm filters (as above) were extracted using the DNeasy^®^ PowerSoil^®^ Pro kit (Qiagen, GmbH, Hilden, Germany) or DNeasy^®^ PowerWater^®^ kit (Qiagen, GmbH, Hilden, Germany), respectively, according to the manufacturer’s instructions. Genomic DNA purity and integrity were determined by using NanoPhotometer^®^ NP80 (IMPLEN, Munich, Germany). DNA was diluted to 1 ng/μL using sterile Mili-Q ddH_2_O, based on the concentration. Amplification of the DNA samples was performed using ITS1F (5′-CTTGGTCATTTAGAGGAAGTAA-3′) and ITS2R (5′-GCTGCGTTCTTCATCGATGC-3′) primers, which target ~500 bp of the fungal rRNA gene internal transcribed spacer (ITS) region. PCR cycling conditions were as follows: 94 °C for 1 min as initial denaturation, 35 cycles of 94 °C for 30 s, 52 °C for 30 s, and 68 °C for 30 s, and a 10 min incubation at 68 °C. The PCR products were purified using GeneJET^TM^ Gel Extraction Kit (Thermo Fisher Scientific, Waltman, MA, USA) and quantified by Nanodrop 2000 (Thermo Fisher Scientific). Amplicon sequencing was performed at the University of Illinois at Chicago (UIC) genome research core for next-generation Illumina Miseq (Illumina, San Diego, CA, USA) sequencing (paired-ended 2 × 300 base sequencing reads). The raw sequences produced in the current study can be accessed through the National Center for Biotechnology (NCBI) under BioProject PRJNA1119211, accession SUB14498584.

### 2.5. Phylogenetic Analysis

Phylogenetic analyses of multiple loci were carried out in order to determine the taxonomic identity of the three cultured isolates from spider cadavers. Sequence data generated from loci from the cultured isolates, as well as sequences obtained from Genbank encompassing representative genera in the family Cordycipitaceae, and the outgroup genus *Trichoderma*, were organized such that sequences for each locus were grouped together into lists of FASTA sequences. Multiple sequence alignments were generated for sequences of each locus individually using the program MAFFT v7.520 [37] with default parameters set. Following alignment, sequences were trimmed using the program GBLOCKS v0.91b [38] with a minimum block length of 3. Following trimming, sequences from the different loci were concatenated using the concat function in the Seqkit v2.4.0 program [39]. Concatenated sequences were used to generate a phylogeny using the program RAxML v8.2.12 [40] with four partitions in the dataset, GTRGAMMA as the model, a rapid bootstrap random number seed of 6669, a parsimony random seed of 2539, and 2000 bootstrap replicates. The hypocrealean fungus, *Trichoderma harzianum*, was used as an outgroup for this tree. To determine how distinct isolate UFSI_5 was from other sequenced *Gibellula* isolates, a second tree was generated using the UFSI_5 ITS sequence and ITS sequences from all available *Gibellula* isolates deposited in genbank. For this tree, sequences were aligned in MAFFT, with no trimming with GBLOCKS to prevent the removal of excessive sequence from the alignment. RAxML was performed as above but without any partitioning of the single locus. For this tree, *Metarrhizium anisopliae* was used as an outgroup. Both trees were visualized using FigTree v1.4.4 [41]. Bootstrap values greater than 70 are displayed on the nodes.

### 2.6. Amplicon Sequence Variants (ASVs) Cluster and Sequence Analyses

Sequences were provided by UIC as fastq files, which were processed through the DADA2 pipeline in the DADA2 R package to assign reads to ASVs using the Majorbio Cloud platform (https://cloud.majorbio.com/, accessed on 15 May 2024) for high-throughput omics data [42]. This process includes filtering and trimming reads with quality scores < 30, constructing an ASV table, removing chimeras, and assigning taxonomy [43]. ASVs were assigned a taxonomic group using the curated fungal database (UNITE) (https://unite.ut.ee/, 20 May 2024) [44], and any ASV with fewer than 2 occurrences was removed [45]. The sampling effectiveness was evaluated with a rarefaction analysis of data subsets using the rank abundance curve (Supplementary Figure S2). Since entomopathogenic fungi (EFs) were considered in this study, only three families of the Hypocreales order and those with the potential of being spider-specific pathogens were chosen to generate the abundance plots, and the rest of the sequences were removed.

### 2.7. Microscopy and Whole-Organism Photography

Following collection, whole infected spiders were imaged using a Zeiss Stemi 305 dissecting microscope (Zeiss, Jena, Germany) and a Keyence VHX digital microscope. On the Keyence VHX digital microscope, automated focus stacking was performed for optimal focus and resolution across the entire surface of spider cadavers. This microscope was also used to image colony growth on Petri dishes. For higher magnification imaging, a Zeiss LSM 800 confocal microscope, a Motic BA310E with a Moticam X3 camera attached (Motic Instruments, Schertz, TX, USA), and a Keyence BZX 800 (Keyence, Itasca, IL, USA) were used. To image the conidiophores, spores, and hyphae from the infected spiders, tissue was carefully removed using a scalpel and forceps and placed onto a drop of water on a microscope slide. A small amount of lactophenol cotton blue was then added to this drop, and the tissue was allowed to sit in the stain for one minute, after which the liquid was wicked away using a Kim Wipe, and a fresh drop of water was added. A cover slip was then added, and the sample was imaged for identifying features using a Keyence BZX 800 and Motic BA310E. To image the morphological features from cultures, a small amount of tissue was removed from plates and wet mounts were prepared as above, but without lactophenol blue staining. The tissue samples were imaged using a Zeiss LSM 800 confocal microscope to image features such as spores, conidiophores, and hyphae. Morphological features were measured using ImageJ.js [46].

## 3. Results

### 3.1. Sampling, Spider Identity, and Isolation from Infected Spiders

**Figure 1:**
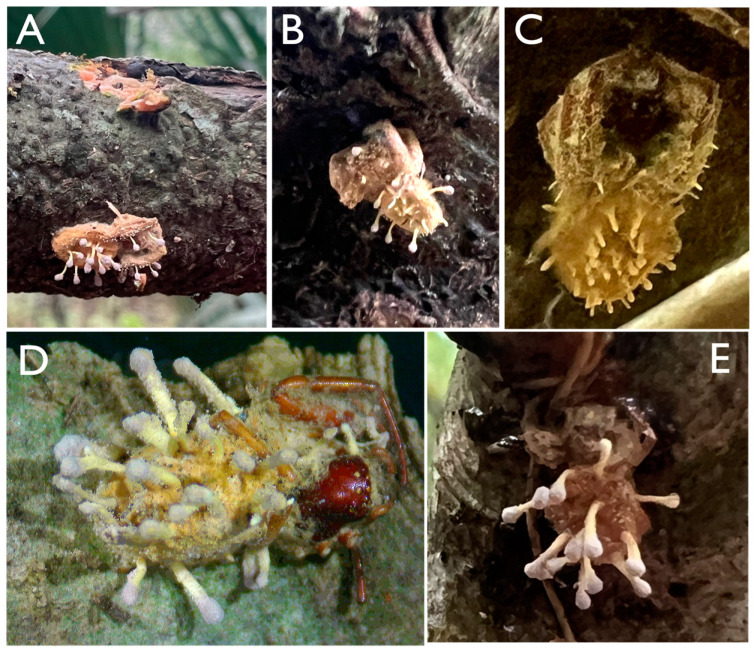
In situ photographs of spider cadavers. (**A**) Spider cadaver S1. (**B**) Spider cadaver S2. (**C**) Spider cadaver S3. (**D**) Spider cadaver S4. (**E**) Spider cadaver S5. Cultivatable fungi were able to be isolated and single-spore purified from cadavers S3–S5 (**C**–**E**).

In total, five infected spiders were located across the two sampling sites, Lake Alice Natural Area and Loblolly Woods, in Gainesville, FL, USA, between the months of March and August 2023. The location of each spider cadaver was noted to be fixed to the bark on the underside of branches and slanted trunks of trees (Figure 1). The position and body arrangement of the spiders all appeared similar, with the front two pairs of legs tucked around the cephalothorax and the abdomen pressed tightly against the tree substrate with a tan to bright yellow mat of hyphae covering most of the arachnid body and affixing it to the tree. Several club-shaped synnemata were observed growing from this mat of hyphae primarily on the abdomen, but also, from the legs and cephalothorax, masses of purple-pigmented conidia were seen forming toward the clubbed ends of these structures. Conidiophores were also noted forming directly from the hyphal mat which covered the body of the spider. Infected spiders were collected using a knife by removing a small piece of the bark to which the cadaver was attached. Spiders were then transported to the lab and stored in sterile, sealed Petri dishes in the dark. Isolations of the infecting fungus were attempted from each of the infected spiders by lightly touching the conidiophores with a sterile inoculating loop and streaking this loop on a PDA plate containing streptomycin and ampicillin for isolation. Using this technique, actively growing cultures were obtained from three of the five infected spiders, S3, S4, and S5, and these cultures were single-spore purified three times to achieve pure cultures as detailed in the Methods section. These isolates were designated UFSI_3, UFSI_4, and UFSI_5, respectively. Based upon the visible morphology of spider hosts (areas not covered by fungal hyphae), including the arrangement of eyes, the color of cephalothorax carapace, and the arrangement of legs, all infected spiders collected appear to belong to the genus *Trachelas* (family Trachelidae).

### 3.2. Growth and Morphology on Different Media Types

**Figure 2:**
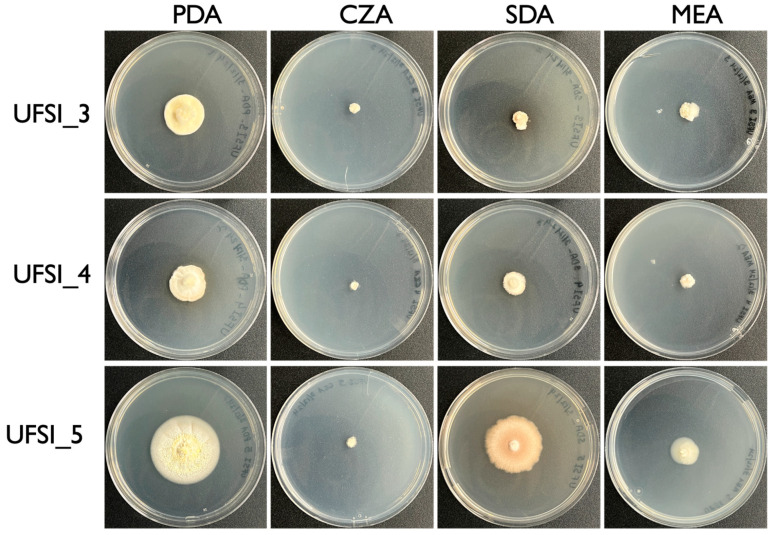
Colony morphology of fungal isolates (UFSI_3, UFSI_4, and UFSI_5) grown on potato dextrose agar (PDA), Czapek–Dox agar (CZA), malt extract agar (MEA), and Sabouraud dextrose agar (SDA) for 30 d at 25 °C.

**Figure 3:**
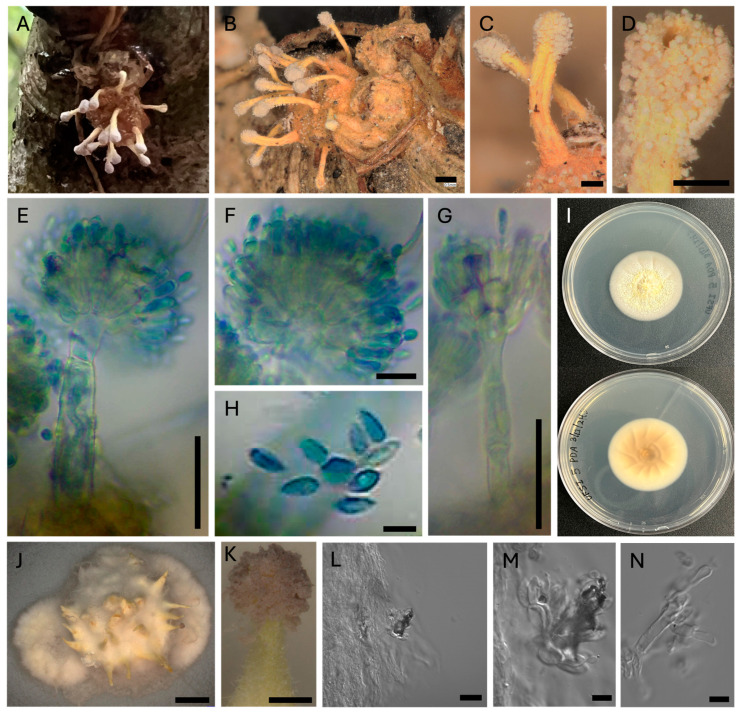
Macroscopic and microscopic morphological characters of *Gibellula* isolate on spider cadavers and in culture. (**A**,**B**) Appearance of spiders (assigned to *Trachelas*) infected by fungal (*Gibellula*) isolates S4 and S5, respectively. (**C**) Synnemata emerging from infected spider cadavers. (**D**) Synnemata head showing purple-pigmented conidiophores. (**E**) Lactophenol blue staining of conidiophore from synnemata showing aspergillus-like morphology. (**F**) Close up of conidiophore head displaying vesicle, metulae, phialides, and conidia. (**G**) Lactophenol blue stained Granulomanus-like asexual morph conidiophore. (**H**) Conidia originating from aspergillus-like asexual morph conidiophores. (**I**) Front and back appearance of *Gibellula* isolate in culture on PDA. (**J**) Gibellula isolate in culture on PDA showing synnema. (**K**) Closeup of synnema produced in culture showing purple-pigmented conidiophores forming at the end. (**L**) Conidiophore forming on synnema produced in culture. (**M**) Closeup of conidiophore produced on synnema in culture. (**N**) Possible Granulomanus-like conidiophore produced in culture. Scale bars in images are as follows: (**A**,**B**) 500 µm, (**C**) 200 µm, (**D**) 200 µm, (**E**) 25 µm, (**F**) 10 µm, (**G**) 25 µm, (**H**) 5 µm, (**J**) 2000 µm, (**K**) 200 µm, (**L**) 20 µm, (**M**) 5 µm, (**N**) 5 µm.

To determine media preference and relative rates of growth, 5 mm plugs of actively growing hyphae from each isolate were cut out of established cultures and placed into the center of plates containing potato dextrose agar (PDA), Czapek–Dox solution agar (CZA), malt extract agar (MEA), and Sabouraud dextrose agar (SDA) and allowed to grow for one month, after which the cultures were examined for their extent of growth and colony morphology (Figure 2). Of the four media types examined, CZA yielded the least amount of growth for all isolates with UFSI_3, UFSI_4, and UFSI_5 showing colony diameters of 7.0, 5.4, and 7.0 mm after 30 d of growth, respectively (Figure 2). Colony morphology on CZA was similar for the three isolates, with dense white mycelia covering the original plug and growing out onto the plate in a thin layer of hyphae. Isolate UFSI_5 showed some small projections growing from the plug on this medium, but no conidiophores or pigmented conidia were noted in any of the plates examined. On the MEA plates, all isolates displayed slightly more robust growth, with UFSI_3, UFSI_4, and UFSI_5 showing 12.9, 9.9, and 18.1 mm colony diameters after 30 d of growth, respectively. On MEA, isolates UFSI_3 and UFSI_4 displayed dense, cottony white to tan mycelia, while the growth of UFSI_5 on this medium appeared tan to yellow, with hyphae forming a much flatter and, in some places, submerged, appearance on plates. On the SDA plates, UFSI_3, UFSI_4, and UFSI_5 grew to a diameter of 9.9, 15.4, and 37.8 mm after 30 d of growth, respectively. The colony morphologies of UFSI_3 and UFSI_4 appeared largely similar, with dense white to tan hyphae spreading from the inoculating plug and growing onto the medium to form a flat colony with irregular edges. A slight darkening of the surrounding medium was noted (Figure 2), suggesting that, under these nutritional conditions, these isolates may be secreting pigmented compounds into the surrounding media. Isolate UFSI_5 displayed a dramatically different morphology on SDA compared to the other isolates as well as to the morphology of UFSI_5 observed on the other media tested. On the SDA, UFSI_5 growth appeared flat to crust-like, with sparse hyphae forming and a submerged irregular appearance, especially at the margins of the colony. The colony displayed an orange to brown coloration which differed from that observed on the other media tested. Of the four media tested, all three isolates displayed the most robust growth on PDA with UFSI_3, UFSI_4, and UFSI_5 showing colony diameters of 25.1, 23.9, and 44.0 mm after 30 d of growth, respectively. On the PDA, isolates UFSI_3 and UFSI_4 displayed largely similar colony morphologies characterized by tan to yellow velvety hyphal growth extending in an irregular margin onto the plate and with some discoloration of the surrounding medium, similar to the discoloration observed on SDA plates. Isolate UFSI_5 grew on PDA as flat to somewhat cottony mycelium with regular margins and a somewhat submerged appearance at the edges of colonies. UFSI_5 on PDA showed dramatic morphological features not observed on other growth media tested, which including the formation of rudimentary synnemata which produced conidiophores and purple-pigmented conidia (Figure 3).

### 3.3. Microscopic Morphology of Fungal Infected Spider Cadavers and in Culture

Morphological structures of the fungal isolate present on spider cadavers S4 and S5, as well as whole spiders, were imaged using dissecting and compound microscopes (Figure 3A,B). Synnemata appeared numerous, yellow fading to tan with age, clavate (Figure 3C,D). The synnemata were curved and were observed to be covered in conidiophores, particularly at their ends, which appeared purple in color. To better view these microscopic structures, synnemata were carefully removed from spider cadavers and stained with lactophenol cotton blue before mounting on microscope slides and imaging (Figure 3E–G). Under a compound microscope, two asexual morphs were noted, *Gibellula*-like asexual morphs which presented aspergillus-like morphologies and Granulomanus-like asexual morphs. The presence of sexual morphs were not noted on any spider cadaver examined. *Gibellula*-like asexual morph conidiophores, vesicles on *Gibellula*-like asexual morphs, metulae, and phialides on conidiophores were observed. The culture grown on PDA appeared off-white to tan, with synnema and purple-pigmented conidiophores visible (Figure 3I–K). Conidiophore forming on synnema produced in culture and possible Granulomanus-like conidiophores produced in culture were noted (Figure 3L–N).

### 3.4. Phylogenetic Placement of Fungal Isolates—Molecular Description of a New Gibellula Species, G. floridensis

**Figure 4:**
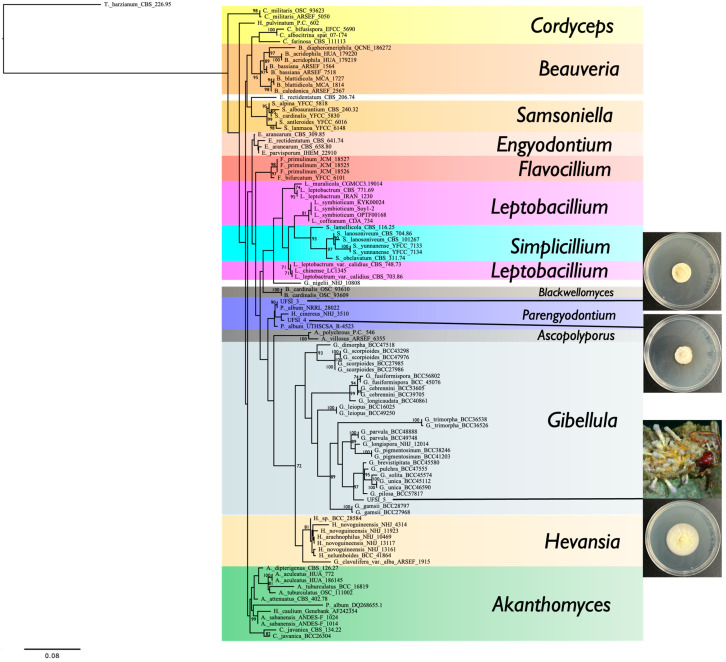
Multilocus maximum likelihood tree of the Cordycipitaceae family generated by RAxML with 2000 bootstrap replicates and containing concatenated ITS, LSU, SSU, and TEF1a sequences to determine the phylogenetic placement of isolates UFSI_3, UFSI_4, and UFSI_5. Bootstrap support values greater than or equal to 70 are displayed on tree nodes.

**Figure 5:**
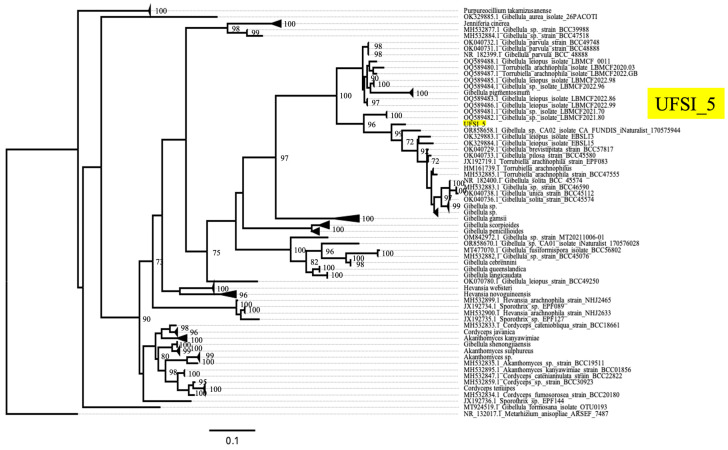
Maximum likelihood ITS tree of UFSI_5 and *Gibellula* sequences downloaded from Genbank. This tree was generated using RAxML with 2000 bootstrap replicates. Bootstrap support values greater than or equal to 70 are displayed on the nodes of the tree. Clades containing many isolates of the same species are collapsed and appear as triangles at the tips of the tree. Isolate UFSI_5 is highlighted in yellow to emphasize its phylogenetic placement among other *Gibellula* species.

To determine the molecular taxonomic identity of the fungal isolates cultured from the infected spider cadavers, genomic DNA was extracted from UFSI_3, UFSI_4, and UFSI_5, and respective ITS, LSU, SSU, and TEF1a loci were amplified and sequenced as detailed in the Methods section (Genbank accession numbers, Supplemental Table S1). Sequences for the above-listed loci corresponding to representative species of the major genera in the family Cordycipitaceae were collected from Genbank, and together with the sequences determined as part of this study, were used to generate a multilocus maximum likelihood phylogenetic tree using RAxML (Figure 4). Following alignment and trimming, 268 bp of ITS, 404 bp of LSU, 237 bp of SSU, and 279 bp of TEF1a were concatenated for a total of 1188 characters used the phylogenetic analysis. In the resulting phylogenetic tree, isolate UFSI_3 grouped closely with the *Parengyodontium album* strain NRRL 28022 with a bootstrap support value of 90%. Isolate UFSI_4 grouped most closely with an isolate labeled as *Hevansia cinnereus* NHJ 3510; however, both isolates together were observed to cluster in the same clade as the above-listed *P. album* isolate and UFSI_3 and between these two isolates and another *Parengyodontium album* strain, UTHSCSA R-4523. This phylogenetic placement, as well as the morphological similarities observed in culture, suggest that the identity of UFSI_4 is likely also *Parengyodontium album* and that the closely clustered *H. cinereus* isolate may represent a *P. album* isolate mislabeled as a species in the genus *Hevansia*. Isolate UFSI_5 clustered within the genus *Gibellula*, with its closest clustering relatives being a clade containing *G. pilosa*, *G. unica*, *G. solita*, *G. pulchra*, and *G. brevistipitata* isolates. However, UFSI_5 formed a distinct branch separate from this clade with a bootstrap support value of 97%, suggesting strong support for this isolate as distinct from these other *Gibellula* species. To better determine the phylogenetic placement of this isolate within a more comprehensive framework of *Gibellula* isolates, a second tree was generated using all available ITS sequences for taxa returned as *Gibellula* in Genbank (Figure 5, Supplementary Table S2). This search yielded a total of 234 sequences, of which 16 were removed after observing preliminary trees due to their abnormally long branch lengths and phylogenetic distance from UFSI_5, indicating that their inclusion was not essential for determining the phylogenetic placement of this isolate. In this *Gibellula*-restricted ITS tree, isolate UFSI_5 cluster with an undescribed *Gibellula* species, *Gibellula* sp. ‘CA02′ isolate CA FUNDIS iNaturalist 170575944., within a clade containing *G. leiopus*, *G. brevistipitata*, *G. pilosa*, *G. solita*, and *G. unica*. However, again, isolate UFSI_5 occupied a neighboring branch which was distinct from this clade with a bootstrap support value of 99%. A comparison of the major morphological characteristics of the isolate with available information for *G. leiopus* and *G. pulchra* was performed (Supplemental Table S3). Given its molecular phylogenetic placements within these two trees coupled to its distinct morphological features and geographical separation from any previously described *Gibellula* species, we identify this *Gibellula* isolate as a new species, *Gibellula floridensis*. Cultures of this *Gibellula* species, as well as of the *P. album* strains UFSI_3 and UFSI_4, have been deposited in the USDA entomopathogenic fungi culture collection ARSEF under the accession numbers 14,792, 14,693, and 14,794, respectively.

### 3.5. Taxonomy

*Gibellula floridensis* sp. nov. R. Joseph and N.O. Keyhani, (Figure 3, Figure 4 and Figure 5).

MycoBank: 856032. 

Etymology: Named after state of Florida where the fungus was collected.

Holotype: USA, Florida, University of Florida, Gainesville 29°39′7.47″ N, 82°19′30.25″ W, from infected Trachelidae spiders, March 2023, R. Joseph (ex-type living culture ARSEF 14792).

Description: Infecting spiders in the family Trachelidae, covering the body with a mat of yellow hyphae, fading to tan with age. Producing synnemata from the abdomen and legs, 438–577 μm long by 46–82 μm wide, yellow, curved, isolated to branching, with clavate ends. Conidiophores bearing purple-pigmented spores forming along synnemata, primarily at ends. *Gibellula*-like asexual morphs of conidiophores 64–100 μm in height and 7–16 μm in width at the widest point of the stalk. Conidial heads measuring 30–50 μm in height and 41–56 μm in width. Vesicles of *Gibellula*-like asexual morphs 11–17 μm in height and 7–9 μm in width. Metulae measuring 8–14μm in height, 3–7 μm in width. Phialides measuring 8–11 μm in length and 2–4 μm in width. Conidia from *Gibellula*-like asexual morphs of conidiophores appearing elongated, curved on one end and tapering to a point at the other end, 5–7 μm in length, 1.5–3 μm in width. Granulomanus morphs of conidiophores measured 54–68 μm in height, 5–7 μm width. Conidial heads measured 29–47 μm in height, 24–40 μm in width. Vesicles measured 9–17 μm in height, 6–8 μm in width. Metulae measured 7–9 μm height, 3–6 μm width. Phialides measuring 7–11 μm length, 2–4 μm width. Conidia measuring 4–7 μm length, 2–4 μm width. No sexual structures were noted on spider cadavers.

Culture characteristics: On potato dextrose agar, cultures appeared white to yellow, with a darkened color visible on the reverse side of the plate and grew to 44.0 mm after 30 d at 25 °C, with colonies displaying flat to somewhat cottony mycelia with regular margins and a somewhat submerged appearance at the edges of colonies, producing sparse, rudimentary synnemata, adorned with conidiophores and purple-pigmented conidia.

Material examined: USA, Florida, University of Florida, Gainesville 42°14′.084″ N, 117°8’.124″ E, from infected Trachelidae spiders, March 2023. Collected specimens destroyed during processing by contaminating fungi, ex-type living culture serving as holotype: ARSEF 14792

Notes: Based on distinct morphological features and multilocus molecular phylogenetic analyses, the strain of the genus *Gibellula* was identified as a new species; nucleotide comparison of ITS, LSU, and *tef1-α* showed differences with the sequences of *Gibellula leiopus* BCC49250, similarities were 80.53% (513/637), 93.4% (726/777), and 94% (695/743). Nucleotide comparisons with the ITS region of *G. leiopus* EBSL13 showed similarities of 93.54% (478/511).

### 3.6. Environmental Sampling of the Fungal Communities Surrounding the Fungal-Infected Cadavers

To address questions concerning the distribution, environmental location/reservoir, and/or occurrence of isolated fungi within the greater fungal community surrounding the infected spiders, samples were collected from four habitat/trophic levels, namely (i) soil (S), (ii) plant litter (PL), (iii) twigs (T), and (iv) leaves (LV) as detailed in the Methods section (outlined in Supplemental Figure S1). Samples were from two locations: (1) Lake Alice (LA) and (2) Loblolly Woods Nature Park (LB) in north-central (Gainesville) Florida. A total of 3 soil, 3 plant litter, 2 twig, and 2 leaf samples were collected from the LA site, with the LB region was divided into four sites. At each of these sites 3 soil, 3 plant litter 1 twig, and 1 leaf samples were collected for a total of 12 soil, 12 plant litter, 4 twig, and 4 leaf samples (for LB). In total, for both LA and LB, 42 samples were collected. Each soil sample consisted of a pool of 5 soil cores along 4 lines (20 cores) in a grid-like pattern as detailed in the Methods section. Each plant litter, twig, and leaf sample consisted of material (~150–200 g) gathered within a ~10 m^2^ area surrounding the infected spiders.

### 3.7. Amplicon-Sequencing Data Output

Environmental samples were processed for ITS amplicon sequencing as detailed in the Methods section. A total of 1,681,793 reads were obtained from the 42 environmental samples; 1,586,552 high-quality reads with an average length of 589 bp were retained after length filtering as well as removal of chimeric sequences. The minimum and maximum read length was 581 to 592 bp, respectively (Supplemental Table S4). Finally, the reads were sorted into samples via barcodes detection/analyses using the global fungal database (UNITE). Data for genera corresponding to insect pathogenic fungi for the families Clavicipitaceae, Cordycipitaceae, and Ophiocordycipitaceae (Hypocrealeas), and other potential specific-spider pathogens were extracted and used for further analysis. This resulted in 30,780 sequence reads corresponding to insect fungal pathogens within these groups with: 18,001 sequence reads found within the Clavicipitaceae, 2160 sequence reads within the Cordycipitaceae, and 9011 sequence reads within the Ophiocordycipitaceae, which were clustered into 112 Amplicon Sequence Variants (ASVs). No sequences corresponding to entomopathogenic fungi belonging to the Entomophthora order were found in our dataset.

### 3.8. Taxonomic Abundance Analysis of EFs

**Figure 6:**
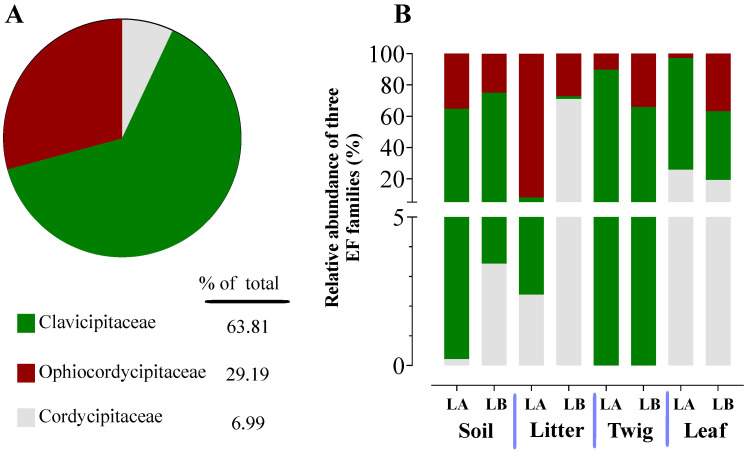
Relative taxonomic abundance of soil-inhabiting entomopathogenic fungi. (**A**) Total percentages of Ophiocordycipitaceae, Clavicipitaceae, and Cordycipitaceae from environmental samples. (**B**) Relative abundances of the three families were detected by amplicon sequencing within each environmental sample type at the two different sampling sites Lake Alice and Loblolly (LA and LB).

The four habitat/trophic types: soils (S), plant litter (PL), twigs (T), and leaves (LV) corresponding to the 42 environmental samples were divided into eight groups, namely Lake Alice (LA-): (1) LAS, (2) LAPL, (3) LAT, and (4) LALV, and Loblolly (LB-): (5) LBS, (6) LBPL, (7) LBT, and (8) LBLV for visualization of the data. The average relative abundance of entomopathogenic fungi from the overall fungal community in terms of the sampling site and habitat revealed was LAS >LBS >LBT > LBLV > LBPL > LAT > LAPL > LALV (representing 6.99, 3.42, 1.73, 0.64, 0.45, 0.14, 0.101, and 0.09% of the total fungal sequences, respectively) (Supplementary Figure S3). Insect–fungal pathogen sequences belonged mainly to Clavicipitaceae (63.81%), followed by Ophiocordycipitaceae (29.19%) and Cordycipitaceae (6.99%) (Figure 6A) across the three families analyzed. Soil, twig, and leaf habitats showed the highest proportion of Clavicipitaceae, whereas plant litter habitats showed a lower proportion of EF families irrespective of sampling locations (Figure 6B).

**Figure 7:**
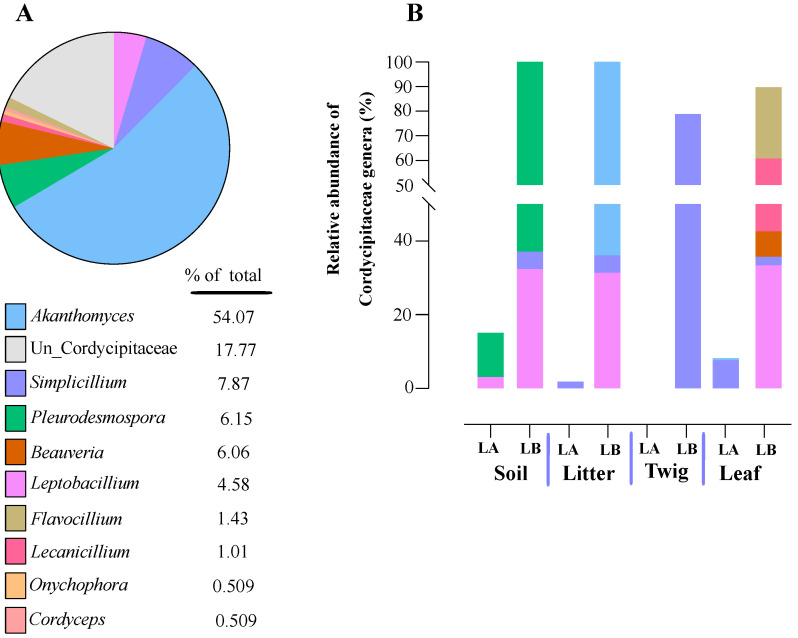
Relative abundance of entomopathogenic fungi in the Cordycipitaceae family from environmental samples. (**A**) Total percentages of Cordycipitaceae genera among all samples. (**B**) Relative abundances of Cordycipitaceae genera from the different environmental samples at both sampling sites (LA and LB).

**Figure 8:**
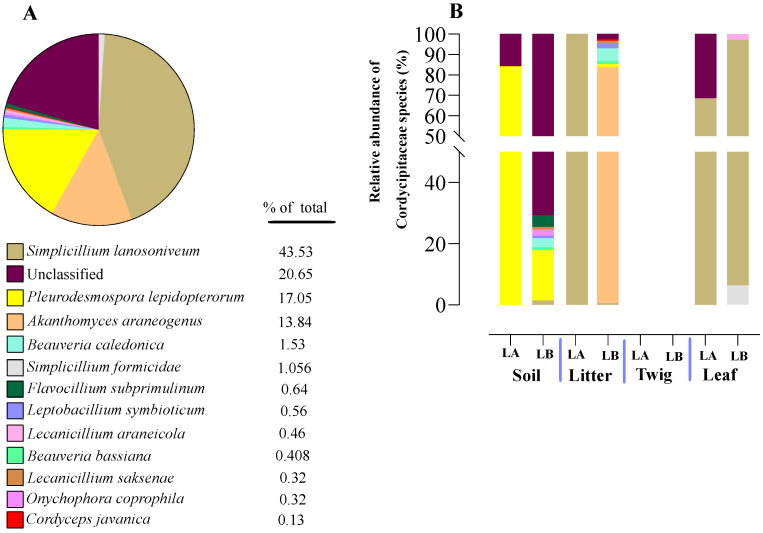
Relative abundance of entomopathogenic fungal species in the family Cordcypitaceae from environmental samples. (**A**) Total percentages of species within the Corcycipitaceae. (**B**) Relative abundances of species within the Cordycipitaceae within environmental sample types and across sample locations (LA and LB).

**Figure 9:**
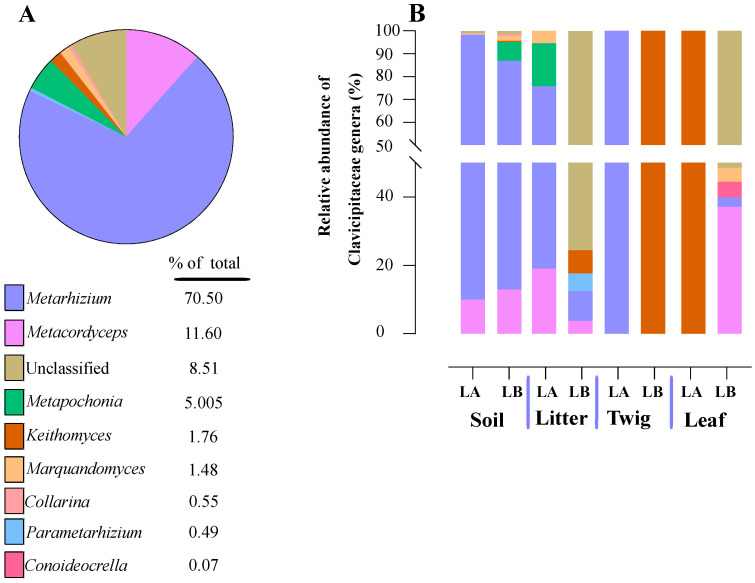
Relative abundance of entomopathogenic fungi in the Clavicipitaceae family from environmental samples. (**A**) Total percentage of Clavicipitaceae genera within all environmental samples. (**B**) Relative abundance of Clavicipitaceae genera for each of the environmental sample types and at the two different sampling locations (LA and LB).

**Figure 10:**
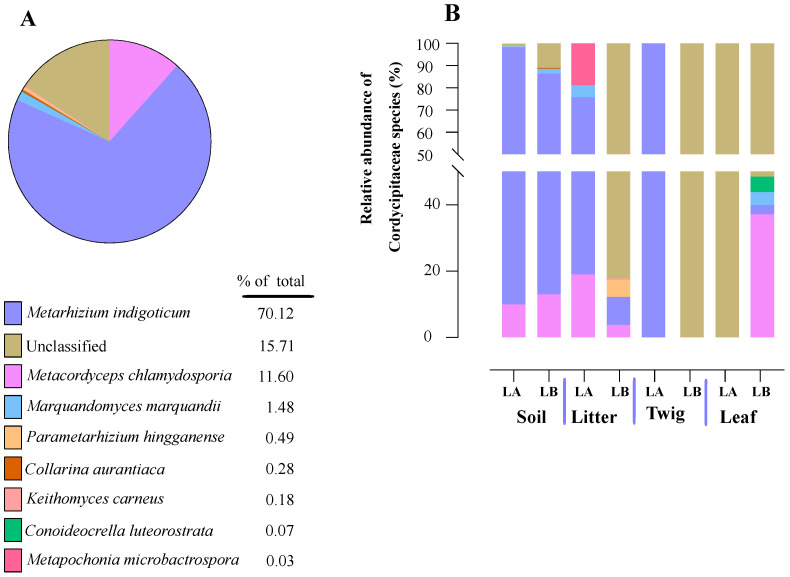
Relative abundances of entomopathogenic fungal species in the Clavicipitaceae from environmental samples. (**A**) Total percentages of Clavicipitaceae fungal species detected in environmental samples. (**B**) Relative abundance of fungal species in the Clavicipitaceae at each environmental sample type and across the two sampling sites (LA and LB).

**Figure 11:**
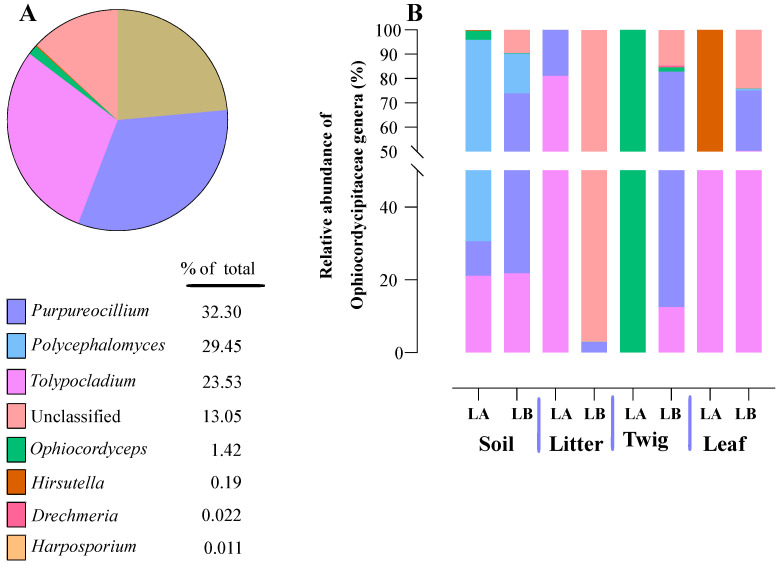
Relative abundances of entomopathogenic fungal genera in the Ophiocordycipitaceae family detected in environmental samples. (**A**) Total percentages of Ophiocordycipitaceae genera detected from environmental samples. (**B**) Relative abundance of Ophiocordycipitaceae genera detected at each environmental sample type from each of the two sampling locations (LA and LB).

**Figure 12:**
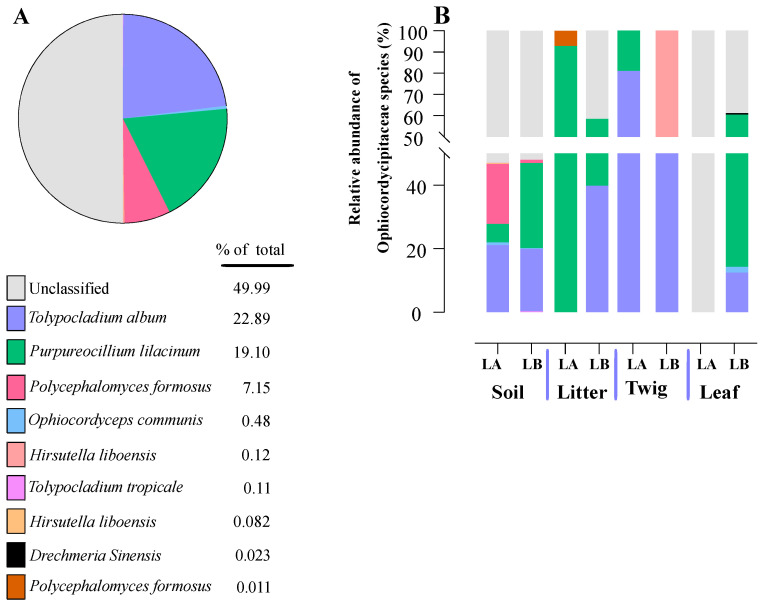
Relative abundances of entomopathogenic fungal species in the Ophiocordycipitaceae detected from environmental samples. (**A**) Total percentages of fungal species in the Ophiocordycipitaceae detected across all environmental samples. (**B**) Relative abundance of fungal species in the Ophiocordycipitaceae detected at each environmental sample type and across the two sampling locations (LA and LB).

Within the Cordycipitaceae family, a total of 9 genera (Figure 7A) and 12 species (Figure 8A) were identified. The genus *Akanthomyces* represented the highest proportion within Cordycipitaceae, accounting for 54.07%, whereas the species *Simplicillium lanosoniveum* was the most prevalent, constituting 43.53% of the species detected. In terms of habitat distribution, the genus *Simplicillium* and the species *S. lanosoniveum* were most abundant on twigs at the Loblolly site (78.82%) and in plant litter at the Lake Alice site (100%), respectively (Figure 7 and Figure 8). In the Clavicipitaceae family, 8 genera (Figure 4) and an equivalent number of species (Figure 9) were observed. Among these, the genus *Metarhizium* showed the highest relative abundance, comprising 70.5% of the detected genera, with *M. indigoticum* being the most dominant species at 70.12%. Notably, both the *Metarhizium* genus and *M. indigoticum* species were found exclusively on twigs at the Lake Alice site, with a 100% occurrence in this habitat type (Figure 9 and Figure 10). The Ophiocordycipitaceae family was represented by 7 genera (Figure 11) and 9 species (Figure 12). Within this family, *Purpureocillium* was the most abundant genus, constituting 32.3% of the total genera detected, while *Tolypocladium album* was the most prevalent species, accounting for 22.89%. The *Ophiocordyceps* genus and *T. album* species exhibited the highest abundance on leaf surfaces (100%) and twigs (81.03%) at the Lake Alice site, respectively (Figure 11 and Figure 12).

### 3.9. Spider-Specific Pathogens in the ITS-Amplicon Dataset

**Figure 13:**
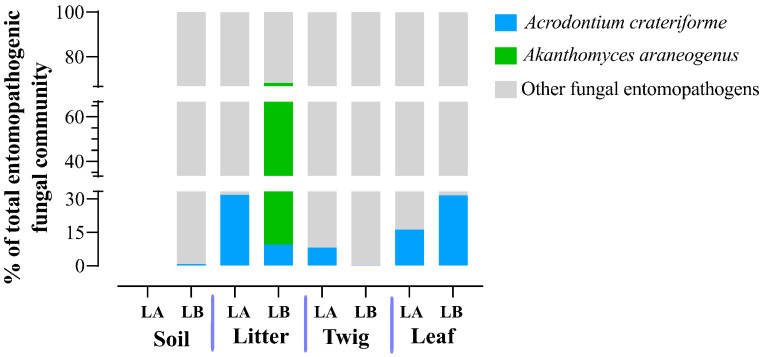
The total percentages of two spider-specific pathogens within the entomopathogenic fungal communities were identified across all environmental sample types and both sampling locations (LA and LB).

Our dataset contained only two fungal pathogens that could be assigned on the species level that are considered specific to spiders: *Acrodontium crateriforme* (Mycosphaerellales, Teratosphaeriaceae) and *Akanthomyces araneogenus* [47]. *Akanthomyces araneogenus* was detected exclusively in plant litter at the Loblolly site, where it constituted 58.62% of the total entomopathogenic fungal community (Figure 13). In contrast, *Acrodontium crateriforme* was found in all environmental samples except soil at the Lake Alice site. The highest proportion (31.74%) of the total entomopathogenic fungal community of *A. crateriforme* was seen in plant litter at Lake Alice. These findings suggest that plant litter may serve as a favorable habitat for these pathogens. No sequences corresponding to *Gibelulla* or *Paraengyodontium* were detected, nor were any sequences found in our ITS dataset for the known major spider fungal pathogens reported in North America [47], including *Akanthomyces lacanii*, *Cordyceps* (*Isaria*/*Paecilomyces farinosus*), *Cordyceps thaxteri*, *Cordyceps* spp., *Hevansia* cf. *aranearum*, *Hevansia* sp., *Ophiocordyceps verrucosa*, *Purpureocillium atypicola* (*Nomuraea atypicola*), *Cladosporium cladosporioides*, *Conidiobolus coronatus*, *Mucor fragilis*, *Sporodiniella umbellata*, and *Cryptococcus depauperatus*.

## 4. Discussion

Interest in insect pathogenic fungi and their related cousins for pest control has garnered significant attention [48,49]; however, thus far, only a relatively small number of spider (Araneae) fungal pathogens have been characterized relative to their insect cousins. Most spider-pathogenic fungi characterized to data are found in the Ascomycota, Hypocreales order [8]. A few generalist, i.e., broad host range, insect fungal pathogens from several genera, e.g., *Beauveria*, *Isaria*, *Hirsutella*, have described species isolated from infected spiders, although the extent to which these represent specialized spider pathogens remains unclear. Members of the *Akanthomyces* genera are typically generalists; however, *A. araneogenus* appears to be spider-specific. The most widely characterized, apparent, and more specific Araneae fungal pathogens are found in the *Gibellula* (sexual morph *Torrubiella*) genera, of which up to 60 species (~30 *Gibellula*, ~28 *Torrubiella*) have been described as capable of infecting at least 25 different spider families.

Here, we identified five spider cadavers apparently infected with fungi in an area of north-central Florida. This region’s climate is defined as humid subtropical, with tropical-like summers, warm to hot shoulder seasons, and mild winters. The area around the sampled regions includes a range of wetlands and aquatic vegetation: grasses, mosses, and other moisture-loving plants, as well as live oaks and palm trees (Lake Alice). The plant canopy in the Loblolly state park zone is mixed hardwood, including loblolly pine (*Pinus taeda*), slash pine (*Pinus elliottii*), laurel oak (*Quercus laurifolia*), water oak (*Quercus nigra*), red maple (*Acer rubrum*), Carolina laurel cherry (*Prunus caroliniana*), sweetgum (*Liquidambar styraciflua*), wax myrtle (*Morella cerifera*), swamp tupelo (*Nyssa biflora*), and sugarberry (*Celtis laevigata*). Based on morphological features and consistent with their range/habitats, the spiders appeared to belong to the genus *Trachelas*. Spider cadavers were found fixed to bark on the underside of branches and trunks, facing downwards. This is consistent with reports of infection mechanisms of above-ground spiders, which normally forage or live close to the ground or soil, by fungal pathogens which result in summit disease, i.e., elevation seeking/negative gravitropism, as the spider host dies. Such behavioral manipulation of the host is considered to maximize spore discharge from the dead spider from heights that would allow for dispersal of infectious spores over a wide area to hosts found in lower trophic levels.

Of the five fungal infected spider cadavers collected, and all had preliminary morphological features consistent with *Gibellula*; however, fungi could only be cultured from three. It is possible that either the fungi on the two specimen from which we could not recovered active fungi were either no longer viable or else fastidious and could not be readily cultured. Of the three fungal isolates that could be cultured and were subsequently single spore purified, two were found to likely represent the same species, and closely matched *Parengyodontium album* NRRL28022 (LSU/SSU 100% match UFSI_3, 98.28% match UFSI_4) and *Parengyodontium album* UTHSCSA_R-4523 (ITS/LSU 99.70% match UFSI_3 and UFSI_4). The third was grouped within *Gibellula*, with its closest match to *G. leiopus* EBSL13 (80% query cover, 93.54% identity) and what has only been listed as *Gibellula* sp. CA02 (ITS, 97% query cover 91.64% identity), albeit both multilocus and greater depth ITS phylogenetic reconstruction indicated the isolate to be distinct. We therefore describe this as a new species, *G. floridensis*, based on combined morphological (Supplementary Table S3) and multilocus molecular phylogenetic characterization. To the best of our knowledge, this is the first description of *Gibellula* from Florida and the broader southeastern United States. Members of the *Gibellula* genera are well known pathogens of a range of spiders [13,14,15,20], and *Gibellula leiopus*/*Gibellula* sp. have been found infecting members of the *Anyphaenidae*, *Araneidae*, *Corinnidae*, *Linyphiidae*, *Pholcidae*, *Salticidae*, *Sparassidae*, *Theridiidae*, *Thomisidae*, and *Zodariidae* families, but not *Trachelidae*, as reported here. For the *Gibellula* sp. isolate that grouped most closely with our isolate, this was found in California, and the host spider identity was not listed [36,50], and these regions are significantly separated from the southeastern US. In addition, multilocus data for *G. leiopus* separated it from *G. floridensis* further than what was seen in the ITS tree, indicating our isolate remains distinct at both the molecular and morphological levels.

*P. album* has a curious distribution and reporting. Originally *Tritirachium* album, then *Beauveria alba*, *Engyodontium album*, it now has its own genera (*Parengyodontium*), with *P. album* as its sole described species, although it is unclear the extent to which various *P. album* isolates have been confirmed via molecular phylogenetic analyses. It is reported to be globally distributed, including from geographically diverse deteriorated (cultural heritage) sites, potentially adapted to salty, moist environments, as well as being an opportunistic mammalian pathogen [51,52,53,54,55,56,57,58,59]. An isolate found on floating plastic debris in the North Pacific Subtropical Gyre has been shown to be able to mineralize plastic [52,60]. *P. album* has been isolated from spider cadavers [61,62,63], but it has not been definitively proven to be a pathogen and not a saprotroph or secondary infection in these cases. Similarly, we cannot make any definitive conclusions concerning whether our isolate is a spider pathogen, and the fact that we isolated *P. album* by performing single-spore isolation from the synnemata and conidiophores from two of the collected spiders was surprising but might point to some kind of secondary association between *P. album* and spider cadavers, or even *P. album* and *Gibellula* species. Additional sampling and/or infection assays are needed to examine this point and/or complete Koch’s postulate.

Knowledge concerning soil fungal communities is an important aspect of understanding effects on forestry and agriculture [64,65,66], and can impact use of entomopathogenic fungi in crop pest protection [67]. An open question concerning facultative, yet specialized insect/arachnid fungal pathogens concerns their environmental distribution (aside from on the host), and at which trophic levels in the environment they can be found. To address this, we performed a systematic sampling of the areas surrounding the spider cadavers, separating our collection into four trophic levels, namely: soil, leaf litter, leaves, and twigs. Sampling was performed following a grid-like pattern that extended the scope to an area of ~10 × 10 m, with multiple sample points for each level that were pooled and subjected to genomic DNA extraction and subsequent amplicon ITS sequencing. Entomopathogenic fungi were found to represent ~1.92% of the total reads generated, and within the three major families, Clavicipitaceae predominated (64%), followed by Ophiocordyceps (29%) and Cordycipitaceae (7%). Important differences between trophic levels were seen, with soil showing the highest diversity, followed by some variation between twigs, leaves, and leaf litter, as well as site (Lake Alice vs. Loblolly nature park). Of note, Cordycipitaceae was found to represent a greater proportion than the others at the leaf trophic level, whereas Clavicipitaceae predominated at the soil and twig levels. For leaf litter, Ophiocordycipitaceae predominated at the Lake Alice site, whereas Cordycipitaceae was found more prevalent in the leaf litter of the Loblolly site. Important differences were also seen at the genera level across the trophic levels and sites, with *Akanthomyces*, *Metarhizium*, *Purpueocillium*, *Tolypocladium*, and *Polycephalomyces* highly abundant. At the species level: *Simplicillium lanosoniveum*, *Pleurodesmonspora lepiopterorum*, and *A. araneogenus* (Cordycipitaceae), *Metarhizium indigoticum* and *Metacordyceps chlamydosporia* (Clavicipitaceae), *Tolypocladium album*, *Purpueocillium lilacinum*, and *Polycephalus formosus* could be identified as highly prevalent. However, it should be noted that a significant portion (9–20%) of our ITS dataset, while categorized at the family level, could not be placed in any described genera, and of those that could be placed into genera, 15–50% could not be assigned at the species level. This indicates significant room for discovery and addition to available databases.

Surprisingly, despite obtaining >1.5 million ITS reads, direct BLAST analysis with the ITS sequences obtained from *G. floridensis* and *P. album*, no hits were obtained. Furthermore, on a broader level, we could not detect any *Gibellula* or *Parengyodontium* even at the genera level in our dataset. There are several potential explanations for these results. First, it cannot be excluded that we did not sample enough, despite examining both areas where the spider cadavers were found and looking at the surrounding soil, leaf litter, leaves, and twigs. Second, our collection excluded insects or other animals in the samples. In tropical areas where spiders are active year-round, it is possible for direct spider to spider transmission of *Gibellula* to account for the full life cycle of this fungus. Another possibility is that other insects can act as a reservoir for the fungus even if they are not hosts, *i.e.*, no disease is caused on these “carriers”. In temperate regions where many species of spider and other arthropod go dormant during the winter, other strategies besides potential spider to spider transmission may be necessary for the fungus to survive this period, including potentially reducing pathogenicity to overwinter with infected individuals or surviving outside of their host in as yet unidentified environmental reservoirs [68]. As Florida is subtropical, direct or carrier-based transmission may prevail, in which case *Gibellula* may be below the detection limit of our sampling. In addition, persistence/survival of *Gibellula* or *Parengyodontium* in the sampled environments may be low. As the cadavers were found after full infection and likely dispersal of any spores, if persistence is low (days to weeks timeframe), the fungi could have been gone by the time we sampled the surrounding environment. We could, however, detect two fungal spider pathogens, *A. crateriforme* and *A. araneogenus* [47]. The latter was found only in the plant litter at the Loblolly but at a high proportion (59%) of the total entomopathogenic fungal community. *A. crateriforme* was widely distributed in all environmental habitats examined except for Lake Alice soil. Wed did not detect any other major spider fungal pathogens described in North America [47]. These data indicate the prevalence of a small subset of spider-specific fungal pathogens in the environment examined. However, some caution should be taken in interpreting any results, and as more species are described and the (ITS) database expands, additional members may be identified. In addition, it is worth noting that we could not detect sequences belonging to members of the Entomophthorales order, which could be due to their lack of presence in our samples but could also be a result of poor efficiency of obtaining such sequences using the ITS amplicon strategy employed which relies on a “universal” primer. Thus, it is possible that ITS amplicon sequencing using such universal primers may poorly target some fungal families and thus bias the data, although this would not affect our ability to detect *Gibellula* or *Parengyodontium*, as these could be readily amplified.

## 5. Conclusions

We have identified a new fungal pathogen of spiders (*Trachelas*), which we have named *Gibellula floridensis* as well as *P. album* from spider cadavers. It is unclear whether the *P. album* represents a true spider pathogen or, as we suspect, an unexamined aspect of secondary associations between *P. album*-spider cadavers or *P. album*-*Gibellula* sp. We further examined the surrounding fungal entomopathogen community across different habitat/trophic levels. To the best of our knowledge, our data represent the first such environmental sampling aimed towards delineating where in the environment these fungi may occur and particularly highlights critical gaps in our understanding of the ecology of more host-specific and/or fastidious insect fungal pathogens. It is recommended that, during future collection and discovery of fungal-infected insects/arachnids, surrounding environmental samples are also collected and analyzed to more broadly begin to address these issues.

## Data Availability

All data are available in the manuscript itself or have been deposited with accession numbers in the appropriate data repositories.

## Supplementary Materials

The following supporting information can be downloaded at: https://www.mdpi.com/article/10.3390/jof10100694/s1, Figure S1: Outline of sample habitats/trophic levels, sample #s and replicates, and geolocation of samples; Figure S2: The rank-abundance curves illustrate the distribution and relative abundance of fungal taxa identified across sampling sites; Figure S3: The proportion of the EF OTUs in comparison with the whole fungal OTU compositions based on percentages; Table S1: Species and Genbank Accession IDs used in construction of multilocus phylogenetic tree; Table S2: Species, isolate codes, Genbank accession IDs, and references for species used in construction of the ITS phylogenetic tree; Table S3: Comparative measurements and morphological characteristics for G. floridensis, G. leiopus, and G. pulchra; Table S4: Overview of raw sequencing data.
