# Supplementary material for: Discovery of Gibellula floridensis from Infected Spiders and Analysis of the Surrounding Fungal Entomopathogen Community
Source: J Fungi (Basel). 2024 Oct 4;10(10):694. doi: 10.3390/jof10100694 (PMC11508666; doi:10.3390/jof10100694)
Supplement: Supplementary file 1 [file jof-10-00694-s001.zip › jof-3211264-supplementary.docx]

**Supplementary Materials**

Discovery of *Gibellula* *floridensis* from infected spiders and analysis of the surrounding fungal entomopathogen community

Ross A. Joseph, Abolfazl Masoudi, Mateo Valdiviezo and Nemat O. Keyhani


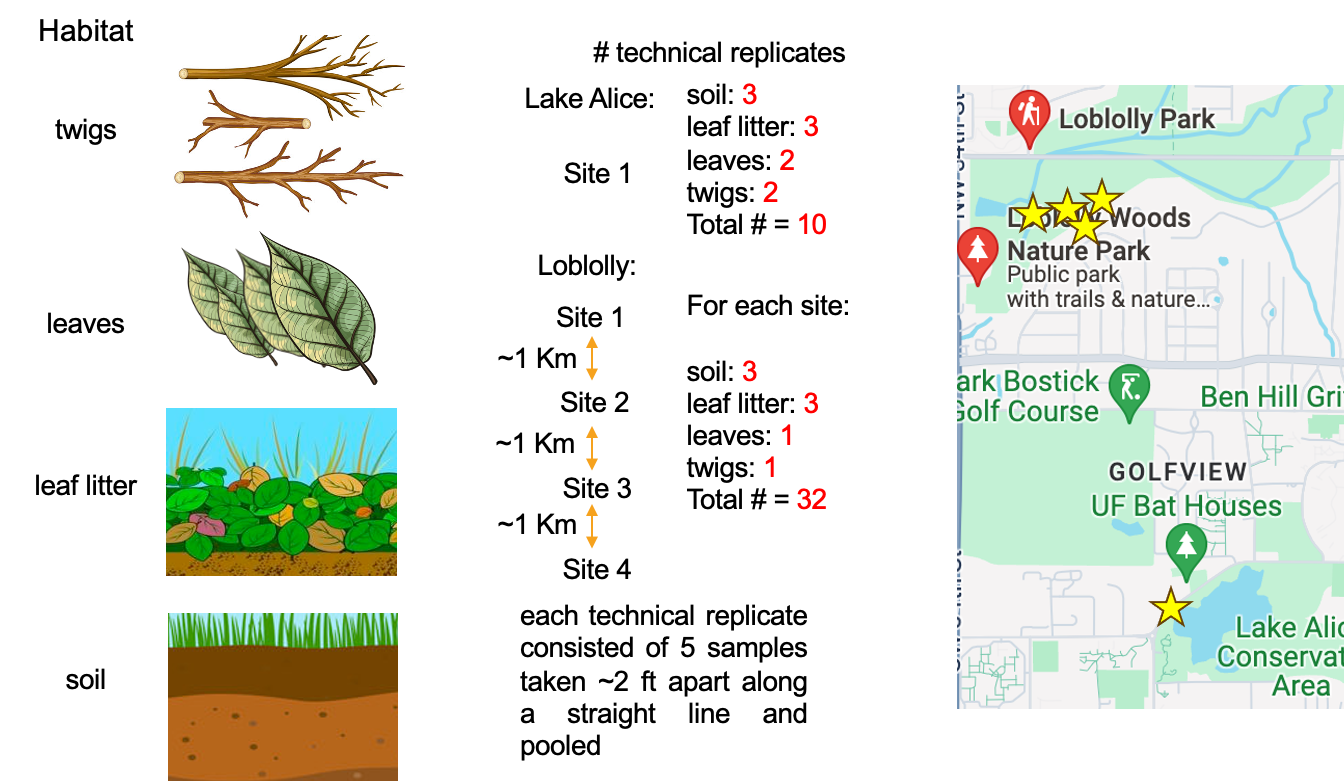


**Supplemental Figure S1.** Outline of sample habitats/trophic levels, sample #s and replicates, and geolocation of sample sites.

**Supplemental Figure S2.** The rank-abundance curves illustrate the distribution and relative abundance of fungal taxa identified across sampling sites. This rank-abundance curve was generated from 42 environmental samples.

**Supplementary Figure S3**. The proportion of the EF OTUs in comparison with the whole fungal OTU compositions based on percentage (A) and the number of sequences (B).

| **Supplemental Table S1.** Species and Genbank Accession IDs used in construction of multilocus phylogenetic tree | | | | | | |
| --- | --- | --- | --- | --- | --- | --- |
| Species | Code | Genbank accession numbers | | | | References |
|  |  | ITS | LSU | SSU | TEF1 |  |
| *A._aculeatus* | HUA_186145 | - | MF416520.1 | MF416572.1 | MF416465.1 | Kepler et. al 2017 |
| *A._aculeatus* | HUA_772 | KC519371.1 | KC519370.1 | KC519368.1 | KC519366.1 | Sanjuan et. al 2014 |
| *A._attenuatus* | CBS_402.78 | AJ292434.1 | AF339565.1 | AF339614.1 | EF468782.1 | Sung et. al 2001 |
| *A._dipterigenus* | CBS_126.27 | OP756342.1 | KM283797.1 | KM283773.1 | KM283820.1 | Manfrino et. al 2022 |
| *A._polychrous* | P.C._546 | - | DQ118737.1 | - | DQ118745.1 | Chaverri et. al 2005 |
| *A._sabanensis* | ANDES-F_1014 | KC633245.1 | KC633248.1 | - | KC875221.1 | Chirivi-Salomon et. al 2013 |
| *A._sabanensis* | ANDES-F_1024 | KC633232.1 | KC875225.1 | - | KC633266.1 | Chiviri-Salomon et. al 2015 |
| *A._tuburculatus* | BCC_16819 | - | MF416546.1 | MF416600.1 | MF416490.1 | Kepler et. al 2017 |
| *A._tuburculatus* | OSC_111002 | JN049830.1 | DQ518767.1 | DQ522553.1 | DQ522338.1 | Spatafora et. al 2007 |
| *A._villosus* | ARSEF_6355 | - | AY886544.1 | - | DQ118750.1 | Chaverri et. al 2005 |
| *B._acridophila* | HUA_179219 | JQ958602.1 | JQ895541.1 | MF416574.1 | JQ958613.1 | Sanjuan et. al 2012 |
| *B._acridophila* | HUA_179220 | JQ958605.1 | JQ895536.1 | JQ895527.1 | JQ958614.1 | Sanjuan et. al 2012 |
| *B._bassiana* | ARSEF_1564 | NR_111594.1 | - | - | HQ880974.1 | Rehner et. al 2011 |
| *B._bassiana* | ARSEF_7518 | HQ880762.1 | - | - | HQ880975.1 | Rehner et. al 2011 |
| *B._blattidicola* | MCA_1727 | - | MF416539.1 | MF416593.1 | MF416483.1 | Kepler et. al 2017 |
| *B._blattidicola* | MCA_1814 | - | MF416540.1 | MF416594.1 | MF416484.1 | Kepler et. al 2017 |
| *B._caledonica* | ARSEF_2567 | HQ880817.1 | AF339520.1 | AF339570.1 | EF469057.1 | Sung et. al 2007 |
| *B._cardinalis* | OSC_93609 | - | AY184962.1 | - | DQ522325.1 | Spatafora et. al 2007 |
| *B._cardinalis* | OSC_93610 | JN049843.1 | AY184963.1 | - | EF469059.1 | Sung et. al 2007 |
| *B._diapheromeriphila* | QCNE_186272 | JQ958608.1 | JQ895534.1 | JQ895530.1 | JQ958610.1 | Sanjuan et. al 2012 |
| *C._albocitrina* | spat_07-174 | - | - | MF416575.1 | MF416467.1 | Kepler et. al 2017 |
| *C._bifusispora* | EFCC_5690 | - | EF468806.1 | EF468952.1 | EF468746.1 | Sung et. al 2007 |
| *C._farinosa* | CBS_111113 | AY624181.1 | MF416554.1 | - | MF416499.1 | Kepler et. al 2017 |
| *C._javanica* | BCC26304 | MH532851.1 | MH394660.1 | - | MH521903.1 | Kuephadungphan 2018 |
| *C._javanica* | CBS_134.22 | NR_111172.1 | NG_059048.1 | - | MF416504.1 | Kepler et. al 2017 |
| *C._militaris* | ARSEF_5050 | HQ880829.1 | - | - | HQ881020.1 | Rehner et. al 2011 |
| *C._militaris* | OSC_93623 | JN049825.1 | AY184966.1 | - | DQ522332.1 | Spatafora et. al 2007 |
| *E._aranearum* | CBS_309.85 | JN036556.1 | AF339526.1 | KM283778.1 | DQ522341.1 | Spatafora et. al 2007 |
| *E._aranearum* | CBS_658.80 | LC092897.1 | LC092916.1 | - | - | Tsang et. al 2016 |
| *E._parvisporum* | IHEM_22910 | LC092896.1 | LC092915.1 | - | LC425558.1 | Lee et. al 2018 |
| *E._rectidentatum* | CBS_206.74 | LC092893.1 | LC092912.1 | - | LC425553.1 | Lee et. al 2018 |
| *E._rectidentatum* | CBS_641.74 | LC092895.1 | LC092914.1 | - | LC425540.1 | Lee et. al 2018 |
| *F._bifurcatum* | YFCC_6101 | MN576833.1 | MN576781.1 | MN576725.1 | MN576951.1 | Wang et. al 2020 |
| *F._primulinum* | JCM_18525 | AB712266.1 | AB712263.1 | NG_073501.1 | LC557125.1 | Higo et. al 2021 |
| *F._primulinum* | JCM_18526 | AB712267.1 | AB712264.1 | - | - | Kaifuchi et. al 2013 |
| *F._primulinum* | JCM_18527 | AB712268.1 | AB712265.1 | - | - | Kaifuchi et. al 2013 |
| *G._brevistipitata* | BCC45580 | OK040729.1 | OK040706.1 | - | OK040697.1 | Kuephadungphan et. al 2022 |
| *G._cebrennini* | BCC39705 | MH532874.1 | MH394673.1 | - | MH521895.1 | Kuephadungphan 2018 |
| *G._cebrennini* | BCC53605 | MT477069.1 | MT477062.1 | - | MT503328.1 | Kuephadungphan et. al 2020 |
| *G._clavulifera_var._alba* | ARSEF_1915 | - | DQ518777.1 | - | DQ522360.1 | Spatafora et. al 2007 |
| *G._dimorpha* | BCC47518 | MH532884.1 | MH394679.1 | - | MH521892.1 | Kuephadungphan 2018 |
| *G._fusiformispora* | BCC_45076 | MH532882.1 | - | - | - | Kuephadungphan 2018 |
| *G._fusiformispora* | BCC56802 | MT477070.1 | MT477063.1 | - | MT503329.1 | Kuephadungphan et. al 2020 |
| *G._gamsii* | BCC27968 | MH152529.1 | MH152539.1 | - | MH152560.1 | Kuephadungphan et. al 2019 |
| *G._gamsii* | BCC28797 | MH152531.1 | MH152541.1 | - | MH152562.1 | Kuephadungphan et. al 2019 |
| *G._leiopus* | BCC16025 | - | MF416548.1 | - | MF416492.1 | Kepler et. al 2017 |
| *G._leiopus* | BCC49250 | OK070780.1 | OK070781.1 | - | OK070782.1 | Kuephadungphan et. al 2022 |
| *G._longicaudata* | BCC40861 | OK040730.1 | OK040707.1 | - | OK040698.1 | Kuephadungphan et. al 2022 |
| *G._longispora* | NHJ_12014 | - | - | - | EU369017.1 | Johnson et. al 2009 |
| *G._nigelii* | NHJ_10808 | - | EU369035.1 | - | EU369018.1 | Johnson et. al 2009 |
| *G._parvula* | BCC48888 | OK040731.1 | OK040708.1 | - | OK040699.1 | Kuephadungphan et. al 2022 |
| *G._parvula* | BCC49748 | OK040732.1 | OK040709.1 | - | OK040700.1 | Kuephadungphan et. al 2022 |
| *G._pigmentosinum* | BCC38246 | MH532872.1 | MH394672.1 | - | MH521893.1 | Kuephadungphan 2018 |
| *G._pigmentosinum* | BCC41203 | MT477071.1 | - | - | MT503330.1 | Kuephadungphan et. al 2020 |
| *G._pilosa* | BCC57817 | OK040733.1 | OK040710.1 | - | OK040701.1 | Kuephadungphan et. al 2022 |
| *G._pulchra* | BCC47555 | MH532885.1 | - | - | MH521897.1 | Kuephadungphan 2018 |
| *G._scorpioides* | BCC27985 | OK040734.1 | MH394662.1 | - | MH521899.1 | Kuephadungphan 2018 |
| *G._scorpioides* | BCC27986 | OK040735.1 | OK040711.1 | - | OK040702.1 | Kuephadungphan et. al 2022 |
| *G._scorpioides* | BCC43298 | MT477074.1 | MH394677.1 | - | MH521900.1 | Kuephadungphan 2018 |
| *G._scorpioides* | BCC47976 | MT477078.1 | MT477066.1 | - | MT503335.1 | Kuephadungphan et. al 2020 |
| *G._solita* | BCC45574 | OK040736.1 | OK040712.1 | - | OK040703.1 | Kuephadungphan et. al 2022 |
| *G._trimorpha* | BCC36526 | OK040737.1 | - | - | OK040704.1 | Kuephadungphan et. al 2022 |
| *G._trimorpha* | BCC36538 | MH532867.1 | MH394668.1 | - | MH521890.1 | Kuephadungphan 2018 |
| *G._unica* | BCC45112 | OK040738.1 | OK040713.1 | - | OK040705.1 | Kuephadungphan et. al 2022 |
| *G._unica* | BCC46590 | MH532883.1 | MH394678.1 | - | - | Kuephadungphan 2018 |
| *H._arachnophilus* | NHJ_10469 | - | EU369031.1 | EU369090.1 | EU369008.1 | Johnson et. al 2009 |
| *H._caulium* | Genebank_AF242354 | - | AF242354.1 | - | - | Sullivan et. al 2000 |
| *H._cinereus* | NHJ_3510 | - | - | EU369091.1 | EU369009.1 | Johnson et. al 2009 |
| *H._nelumboides* | BCC_41864 | JN201871.1 | JN201873.1 | JN201863.1 | JN201867.1 | Thanakitpipattana et. al 2011 |
| *H._novoguineensis* | NHJ_11923 | - | EU369032.1 | EU369095.1 | EU369013.1 | Johnson et. al 2009 |
| *H._novoguineensis* | NHJ_13117 | - | - | EU369092.1 | EU369010.1 | Johnson et. al 2009 |
| *H._novoguineensis* | NHJ_13161 | - | - | EU369093.1 | EU369011.1 | Johnson et. al 2007 |
| *H._novoguineensis* | NHJ_4314 | - | - | EU369094.1 | EU369012.1 | Johnson et. al 2009 |
| *H._pulvinatum* | P.C._602 | - | DQ118738.1 | - | DQ118746.1 | Chaverri et. al 2005 |
| *H._sp.* | BCC_28584 | - | GQ249989.1 | GQ249965.1 | GQ250040.1 | Ridkaew et. al 2009 |
| *L._chinense* | LC1345 | JQ410324.1 | JQ410322.1 | - | - | Liu and Cai 2012 |
| *L._coffeanum* | CDA_734 | MF066034.1 | MF066032.1 | - | - | Gomes et. al 2018 |
| *L._leptobactrum* | CBS_771.69 | EF641868.1 | KU382224.1 | EF641852.1 | - | Zare and Gams 2007 |
| *L._leptobactrum* | IRAN_1230 | - | KU382225.1 | - | - | Zare and Gams 2016 |
| *L._leptobactrum_var._calidius* | CBS_703.86 | EF641866.1 | KU382226.1 | - | - | Zare and Gams 2016 |
| *L._leptobactrum_var._calidius* | CBS_748.73 | OW985233.1 | KU382227.1 | - | - | Zare and Gams 2016 |
| *L._muralicola* | CGMCC3.19014 | MH379983.1 | MH379997.1 | - | - | Sun et. al 2018 |
| *L._symbioticum* | KYK00024 | - | AB378539.1 | - | - | Kurihara 2008 |
| *L._symbioticum* | OPTF00168 | - | LC506047.1 | - | - | Okane et. al 2020 |
| *L._symbioticum* | Soy1-2 | - | LC506046.1 | - | - | Okane et. al 2020 |
| *P._album* | DQ268655.1 | - | - | - | DQ268655.1 | Jasmin et. al 2005 |
| *P._album* | NRRL_28022 | - | AF049167.1 | AF049147.1 | - | Cigelnik 1998 |
| *P._album* | UTHSCSA_R-4523 | HM214540.1 | HM214541.1 | - | - | Balasingham et. al 2011 |
| *S._alboaurantium* | CBS_240.32 | MH855305.1 | JF415979.1 | JF415958.1 | JF416019.1 | Kepler et. al 2012 |
| *S._alpina* | YFCC_5818 | OQ476469.1 | MN576809.1 | MN576753.1 | MN576979.1 | Wang et. al 2020 |
| *S._antleroides* | YFCC_6016 | OQ476471.1 | MN576803.1 | MN576747.1 | MN576973.1 | Wang et. al 2020 |
| *S._cardinalis* | YFCC_5830 | OQ476478.1 | MN576788.1 | MN576732.1 | MN576958.1 | Wang et. al 2020 |
| *S._lamellicola* | CBS_116.25 | MH854806.1 | AF339552.1 | AF339601.1 | DQ522356.1 | Spatafora et. al 2007 |
| *S._lanmaoa* | YFCC_6148 | OQ476485.1 | MN576789.1 | MN576733.1 | MN576959.1 | Wang et. al 2020 |
| *S._lanosoniveum* | CBS_101267 | - | AF339554.1 | AF339603.1 | DQ522357.1 | Spatafora et. al 2007 |
| *S._lanosoniveum* | CBS_704.86 | AJ292396.1 | AF339553.1 | AF339602.1 | DQ522358.1 | Spatafora et. al 2007 |
| *S._obclavatum* | CBS_311.74 | MH860859.1 | AF339517.1 | AF339567.1 | EF468798.1 | Sung et. al 2007 |
| *S._yunnanense* | YFCC_7133 | - | MN576784.1 | MN576728.1 | MN576954.1 | Wang et. al 2020 |
| *S._yunnanense* | YFCC_7134 | - | MN576785.1 | MN576729.1 | MN576955.1 | Wang et. al 2020 |
| *T._harzianum* | CBS_226.95 | AY605713.1 | MH874152.1 | AF276094.1 | AF348101.1 | Samuels et. al 2002 |
| *P. album* | UFSI_3 | PP915745 | PP915999 | PP916007 | - | **This study** |
| *P. album* | UFSI_4 | PP915746 | PP916000 | PP916008 | - | **This study** |
| *G. floridensis* | UFSI_5 | PP915747 | PP916001 | PP916009 | PP938454 | **This study** |

| **Supplemental Table S2**. Species, isolate codes, Genbank accession IDs, and references for species used in construction of the ITS phylogenetic tree. | | | |
| --- | --- | --- | --- |
| Species | Code | Genbank accession | Reference |
| *Gibellula gamsii* | BCC27968 | MH152529.1 | Kuephadungphan et. al 2019 |
| *Gibellula gamsii* | EPF034 | JX192720.1 | Kuephadungphan et. al 2019 |
| *Gibellula gamsii* | BCC42026 | MH152537.1 | Kuephadungphan et. al 2019 |
| *Gibellula gamsii* | BCC30397 | MH152536.1 | Kuephadungphan et. al 2019 |
| *Gibellula gamsii* | BCC30396 | MH152535.1 | Kuephadungphan et. al 2019 |
| *Gibellula gamsii* | BCC30449 | MH152534.1 | Kuephadungphan et. al 2019 |
| *Gibellula gamsii* | BCC29228 | MH152533.1 | Kuephadungphan et. al 2019 |
| *Gibellula gamsii* | BCC25798 | MH152532.1 | Kuephadungphan et. al 2019 |
| *Gibellula gamsii* | BCC28797 | MH152531.1 | Kuephadungphan et. al 2019 |
| *Gibellula gamsii* | BCC27970 | MH152530.1 | Kuephadungphan et. al 2019 |
| *Gibellula sp. WK-2013* | EPF163 | JX192733.1 | Kuephadungphan et. al 2012 |
| *Gibellula sp. WK-2013* | EPF157 | JX192732.1 | Kuephadungphan et. al 2012 |
| *Gibellula sp. WK-2013* | EPF150 | JX192731.1 | Kuephadungphan et. al 2012 |
| *Gibellula sp. WK-2013* | EPF149 | JX192730.1 | Kuephadungphan et. al 2012 |
| *Gibellula sp. WK-2013* | EPF147 | JX192729.1 | Kuephadungphan et. al 2012 |
| *Gibellula sp. WK-2013* | EPF136 | JX192728.1 | Kuephadungphan et. al 2012 |
| *Gibellula sp. WK-2013* | EPF135 | JX192727.1 | Kuephadungphan et. al 2012 |
| *Gibellula sp. WK-2013* | EPF124 | JX192726.1 | Kuephadungphan et. al 2012 |
| *Gibellula sp. WK-2013* | EPF120 | JX192725.1 | Kuephadungphan et. al 2012 |
| *Gibellula sp. WK-2013* | EPF106 | JX192724.1 | Kuephadungphan et. al 2012 |
| *Gibellula sp. WK-2013* | EPF081 | JX192723.1 | Kuephadungphan et. al 2012 |
| *Gibellula sp. WK-2013* | EPF080 | JX192722.1 | Kuephadungphan et. al 2012 |
| *Gibellula sp. WK-2013* | EPF079 | JX192721.1 | Kuephadungphan et. al 2012 |
| *Gibellula sp. WK-2013* | EPF004 | JX192718.1 | Kuephadungphan et. al 2012 |
| *Gibellula leiopus* |  | KP685597.1 | Liang et. al 2015 |
| *Gibellula clavulifera var. clavulifera* |  | KP685596.1 | Liang et. al 2015 |
| *Gibellula sp.* | BCC14505 | GQ250021.1 | Ridkaew et. al 2009 |
| *Gibellula sp.* | BCC02757 | GQ250020.1 | Ridkaew et. al 2009 |
| *Gibellula clavispora* | C07815 | KJ857270.1 | Liang et. al 2014 |
| *Gibellula clavulifera var. clavulifera* | GZUIFR-HN0801 | KJ857269.1 | Liang et. al 2014 |
| *Gibellula sp.* | MY2566.01 | HM161738.1 | Ridkaew et. al 2010 |
| *Gibellula sp.* | NHJ 13158 | JN049864.1 | Kepler et. al 2011 |
| *Gibellula trimorpha* | BCC36526 | OK040737.1 | Kuephadungphan et. al 2022 |
| *Gibellula solita* | BCC45574 | OK040736.1 | Kuephadungphan et. al 2022 |
| *Gibellula parvula* | BCC48888 | OK040731.1 | Kuephadungphan et. al 2022 |
| *Gibellula longicaudata* | BCC40861 | OK040730.1 | Kuephadungphan et. al 2022 |
| *Gibellula aurea* | 26PACOTI | OK329885.1 | Mendes-Pereira et. al, 2021 |
| *Gibellula penicillioides* | GNJ20200814-14 | MW969670.1 | Wang and Chen 2021 |
| *Gibellula pigmentosinum* | BCC41203 | MT477071.1 | Kuephadungphan et. al 2020 |
| *Gibellula fusiformispora* | BCC56802 | MT477070.1 | Kuephadungphan et. al 2020 |
| *Gibellula shennongjiaensis* | GZAC-SNJ2012 | KT728708.1 | Zou et. al 2015 |
| *Gibellula queenslandica* | BRIP 72767a | OR452099.1 | Tan and Shivas 2023 |
| *Gibellula leiopus* | LBMCF_0011 | OQ589488.1 | Mendes-Pereira et. al, 2023 |
| *Gibellula leiopus* | LBMCF2022.99 | OQ589486.1 | Mendes-Pereira et. al, 2023 |
| *Gibellula leiopus* | LBMCF2022.98 | OQ589485.1 | Mendes-Pereira et. al, 2023 |
| *Gibellula sp.* | LBMCF2022.96 | OQ589484.1 | Mendes-Pereira et. al, 2023 |
| *Gibellula leiopus* | LBMCF2022.86 | OQ589483.1 | Mendes-Pereira et. al, 2023 |
| *Gibellula sp.* | LBMCF2021.80 | OQ589482.1 | Mendes-Pereira et. al, 2023 |
| *Gibellula sp.* | LBMCF2021.70 | OQ589481.1 | Mendes-Pereira et. al, 2023 |
| *Gibellula sp.* | LBMCF2020.01 | OQ589479.1 | Mendes-Pereira et. al, 2023 |
| *Gibellula leiopus* | BCC49250 | OK070780.1 | Kuephadungphan et. al 2022 |
| *Gibellula unica* | BCC45112 | OK040738.1 | Kuephadungphan et. al 2022 |
| *Gibellula scorpioides* | BCC27986 | OK040735.1 | Kuephadungphan et. al 2022 |
| *Gibellula scorpioides* | BCC27985 | OK040734.1 | Kuephadungphan et. al 2022 |
| *Gibellula pilosa* | BCC45580 | OK040733.1 | Kuephadungphan et. al 2022 |
| *Gibellula parvula* | BCC49748 | OK040732.1 | Kuephadungphan et. al 2022 |
| *Gibellula brevistipitata* | BCC57817 | OK040729.1 | Kuephadungphan et. al 2022 |
| *Gibellula leiopus* | EBSL15 | OK329884.1 | Mendes-Pereira et. al, 2021 |
| *Gibellula leiopus* | EBSL13 | OK329883.1 | Mendes-Pereira et. al, 2021 |
| *Gibellula penicillioides* | GNJ20200812-05 | MW969672.1 | Wang and Chen 2021 |
| *Gibellula penicillioides* | GNJ20200814-17 | MW969671.1 | Wang and Chen 2021 |
| *Gibellula penicillioides* | GNJ20200814-11 | MW969669.1 | Wang and Chen 2021 |
| *Gibellula scorpioides* | BCC47976 | MT477078.1 | Kuephadungphan et. al 2020 |
| *Gibellula scorpioides* | BCC47530 | MT477077.1 | Kuephadungphan et. al 2020 |
| *Gibellula scorpioides* | BCC47514 | MT477076.1 | Kuephadungphan et. al 2020 |
| *Gibellula scorpioides* | BCC45127 | MT477075.1 | Kuephadungphan et. al 2020 |
| *Gibellula scorpioides* | BCC43298 | MT477074.1 | Kuephadungphan et. al 2020 |
| *Gibellula scorpioides* | BCC13020 | MT477073.1 | Kuephadungphan et. al 2020 |
| *Gibellula pigmentosinum* | BCC41870 | MT477072.1 | Kuephadungphan et. al 2020 |
| *Gibellula cebrennini* | BCC53605 | MT477069.1 | Kuephadungphan et. al 2020 |
| *Gibellula cebrennini* | BCC53551 | MT477068.1 | Kuephadungphan et. al 2020 |
| *Gibellula cebrennini* | BCC32072 | MT477067.1 | Kuephadungphan et. al 2020 |
| *Gibellula sp.* | BCC47518 | MH532884.1 | Kuephadungphan 2018 |
| *Gibellula sp.* | BCC46590 | MH532883.1 | Kuephadungphan 2018 |
| *Gibellula sp.* | BCC45076 | MH532882.1 | Kuephadungphan 2018 |
| *Gibellula sp.* | BCC42046 | MH532879.1 | Kuephadungphan 2018 |
| *Gibellula sp.* | BCC41184 | MH532878.1 | Kuephadungphan 2018 |
| *Gibellula sp.* | BCC39988 | MH532877.1 | Kuephadungphan 2018 |
| *Gibellula sp.* | BCC39709 | MH532876.1 | Kuephadungphan 2018 |
| *Gibellula sp.* | BCC39707 | MH532875.1 | Kuephadungphan 2018 |
| *Gibellula sp.* | BCC39705 | MH532874.1 | Kuephadungphan 2018 |
| *Gibellula sp.* | BCC39007 | MH532873.1 | Kuephadungphan 2018 |
| *Gibellula sp.* | BCC38246 | MH532872.1 | Kuephadungphan 2018 |
| *Gibellula sp.* | BCC37860 | MH532871.1 | Kuephadungphan 2018 |
| *Gibellula sp.* | BCC37632 | MH532870.1 | Kuephadungphan 2018 |
| *Gibellula sp.* | BCC36538 | MH532867.1 | Kuephadungphan 2018 |
| *Gibellula sp.* | BCC27981 | MH532852.1 | Kuephadungphan 2018 |
| *Gibellula curvispora* | GZUIFR1.1 | JQ342826.1 | Han et. al 2011 |
| *Gibellula sp. 'CA01'* | iNaturalist 170576028 | OR858670.1 | D'Elia et. al 2023 |
| *Gibellula sp. 'CA02'* | CA FUNDIS iNaturalist 170575944 | OR858658.1 | D'Elia et. al 2023 |
| *Gibellula gamsii* | MFLU:22-0275 | OQ127354.1 | Wei et. al 2022 |
| *Gibellula sp.* | MES-3844 | ON383415.1 | Smith and Kaminsky 2022 |
| *Gibellula sp. TW-2021a* | NL20210822-20 | OL982303.1 | Chen 2021 |
| *Gibellula sp. TW-2021a* | NL20210822-09 | OL982302.1 | Chen 2021 |
| *Gibellula sp. TW-2021a* | NL20210822-01 | OL982301.1 | Chen 2021 |
| *Gibellula sp.* | MT20211006-01 | OM842972.1 | Wang and Chen 2022 |
| *Gibellula formosana* | OTU0193 | MT924519.1 | Xiang 2020 |
| *Uncultured Gibellula* | OTU0398 | MT924724.1 | Xiang 2020 |
| *Torrubiella arachnophila* | EPF083 | JX192719.1 | Kuephadungphan et. al 2012 |
| *Torrubiella arachnophilus* |  | KP685595.1 | Liang et. al 2015 |
| *Torrubiella arachnophilus* | GZUIFR-SL120905 | KJ857268.1 | Liang et. al 2014 |
| *Torrubiella arachnophilus* |  | HM161739.1 | Ridkaew et. al 2010 |
| *Akanthomyces websteri* | BCC23860 | GQ250009.1 | Kuephadungphan et. al 2019 |
| *Akanthomyces cinereus* | BCC02191 | GQ250000.1 | Kuephadungphan et. al 2019 |
| *Sporothrix sp.* | EPF144 | JX192736.1 | Kuephadungphan et. al 2012 |
| *Sporothrix sp.* | EPF127 | JX192735.1 | Kuephadungphan et. al 2012 |
| *Sporothrix sp.* | EPF089 | JX192734.1 | Kuephadungphan et. al 2012 |
| *Jenniferia cinerea* | EPF156 | JX192717.1 | Kuephadungphan et. al 2012 |
| *Jenniferia cinerea* | EPF155 | JX192716.1 | Kuephadungphan et. al 2012 |
| *Jenniferia cinerea* | EPF128 | JX192715.1 | Kuephadungphan et. al 2012 |
| *Jenniferia cinerea* | EPF094 | JX192714.1 | Kuephadungphan et. al 2012 |
| *Jenniferia cinerea* | EPF056 | JX192713.1 | Kuephadungphan et. al 2012 |
| *Hevansia novoguineensis* | EPF161 | JX192712.1 | Kuephadungphan et. al 2012 |
| *Hevansia novoguineensis* | EPF160 | JX192711.1 | Kuephadungphan et. al 2012 |
| *Hevansia novoguineensis* | EPF146 | JX192710.1 | Kuephadungphan et. al 2012 |
| *Hevansia novoguineensis* | EPF145 | JX192709.1 | Kuephadungphan et. al 2012 |
| *Hevansia novoguineensis* | EPF143 | JX192708.1 | Kuephadungphan et. al 2012 |
| *Hevansia novoguineensis* | EPF142 | JX192707.1 | Kuephadungphan et. al 2012 |
| *Hevansia novoguineensis* | EPF141 | JX192706.1 | Kuephadungphan et. al 2012 |
| *Hevansia novoguineensis* | EPF140 | JX192705.1 | Kuephadungphan et. al 2012 |
| *Hevansia novoguineensis* | EPF133 | JX192704.1 | Kuephadungphan et. al 2012 |
| *Hevansia novoguineensis* | EPF132 | JX192703.1 | Kuephadungphan et. al 2012 |
| *Hevansia novoguineensis* | EPF131 | JX192702.1 | Kuephadungphan et. al 2012 |
| *Hevansia novoguineensis* | EPF126 | JX192701.1 | Kuephadungphan et. al 2012 |
| *Hevansia novoguineensis* | EPF125 | JX192700.1 | Kuephadungphan et. al 2012 |
| *Hevansia novoguineensis* | EPF111 | JX192699.1 | Kuephadungphan et. al 2012 |
| *Hevansia novoguineensis* | EPF110 | JX192698.1 | Kuephadungphan et. al 2012 |
| *Hevansia novoguineensis* | EPF108 | JX192697.1 | Kuephadungphan et. al 2012 |
| *Hevansia novoguineensis* | EPF104 | JX192696.1 | Kuephadungphan et. al 2012 |
| *Hevansia novoguineensis* | EPF101 | JX192695.1 | Kuephadungphan et. al 2012 |
| *Hevansia novoguineensis* | EPF100 | JX192694.1 | Kuephadungphan et. al 2012 |
| *Hevansia novoguineensis* | EPF099 | JX192693.1 | Kuephadungphan et. al 2012 |
| *Hevansia novoguineensis* | EPF098 | JX192692.1 | Kuephadungphan et. al 2012 |
| *Hevansia novoguineensis* | EPF097 | JX192691.1 | Kuephadungphan et. al 2012 |
| *Hevansia novoguineensis* | EPF093 | JX192690.1 | Kuephadungphan et. al 2012 |
| *Hevansia novoguineensis* | EPF092 | JX192689.1 | Kuephadungphan et. al 2012 |
| *Hevansia novoguineensis* | EPF076 | JX192688.1 | Kuephadungphan et. al 2012 |
| *Hevansia novoguineensis* | EPF075 | JX192687.1 | Kuephadungphan et. al 2012 |
| *Hevansia novoguineensis* | EPF074 | JX192686.1 | Kuephadungphan et. al 2012 |
| *Hevansia novoguineensis* | EPF071 | JX192685.1 | Kuephadungphan et. al 2012 |
| *Hevansia novoguineensis* | EPF070 | JX192684.1 | Kuephadungphan et. al 2012 |
| *Hevansia novoguineensis* | EPF068 | JX192683.1 | Kuephadungphan et. al 2012 |
| *Hevansia novoguineensis* | EPF063 | JX192682.1 | Kuephadungphan et. al 2012 |
| *Hevansia novoguineensis* | EPF057 | JX192681.1 | Kuephadungphan et. al 2012 |
| *Hevansia novoguineensis* | EPF047 | JX192680.1 | Kuephadungphan et. al 2012 |
| *Hevansia novoguineensis* | EPF046 | JX192679.1 | Kuephadungphan et. al 2012 |
| *Hevansia novoguineensis* | EPF041 | JX192678.1 | Kuephadungphan et. al 2012 |
| *Hevansia novoguineensis* | EPF037 | JX192677.1 | Kuephadungphan et. al 2012 |
| *Hevansia novoguineensis* | EPF036 | JX192676.1 | Kuephadungphan et. al 2012 |
| *Hevansia novoguineensis* | EPF021 | JX192675.1 | Kuephadungphan et. al 2012 |
| *Hevansia novoguineensis* | EPF020 | JX192674.1 | Kuephadungphan et. al 2012 |
| *Hevansia novoguineensis* | EPF019 | JX192673.1 | Kuephadungphan et. al 2012 |
| *Torrubiella arachnophila* | LBMCF2022.GB | OQ589487.1 | Mendes-Pereira et. al, 2023 |
| *Torrubiella arachnophila* | LBMCF2020.03 | OQ589480.1 | Mendes-Pereira et. al, 2023 |
| *Torrubiella arachnophila* | BCC47555 | MH532885.1 | Kuephadungphan 2018 |
| *Hevansia arachnophila* | NHJ2633 | MH532900.1 | Kuephadungphan 2018 |
| *Hevansia arachnophila* | NHJ2465 | MH532899.1 | Kuephadungphan 2018 |
| *Akanthomyces kanyawimiae* | BCC15619 | MH532898.1 | Kuephadungphan 2018 |
| *Hevansia novoguineensis* | BCC09740 | MH532897.1 | Kuephadungphan 2018 |
| *Jenniferia cinerea* | BCC02191 | MH532896.1 | Kuephadungphan 2018 |
| *Akanthomyces kanyawimiae* | BCC01856 | MH532895.1 | Kuephadungphan 2018 |
| *Akanthomyces kanyawimiae* | BCC01633 | MH532894.1 | Kuephadungphan 2018 |
| *Cordyceps javanica* | BCC01857 | MH532893.1 | Kuephadungphan 2018 |
| *Cordyceps javanica* | BCC01840 | MH532892.1 | Kuephadungphan 2018 |
| *Purpureocillium takamizusanense* | BCC66197 | MH532891.1 | Kuephadungphan 2018 |
| *Purpureocillium takamizusanense* | BCC66196 | MH532890.1 | Kuephadungphan 2018 |
| *Purpureocillium takamizusanense* | BCC66195 | MH532889.1 | Kuephadungphan 2018 |
| *Purpureocillium takamizusanense* | BCC66194 | MH532888.1 | Kuephadungphan 2018 |
| *Purpureocillium takamizusanense* | BCC66193 | MH532887.1 | Kuephadungphan 2018 |
| *Akanthomyces sulphureus* | BCC47986 | MH532886.1 | Kuephadungphan 2018 |
| *Hevansia novoguineensis* | BCC44251 | MH532881.1 | Kuephadungphan 2018 |
| *Jenniferia cinerea* | BCC42062 | MH532880.1 | Kuephadungphan 2018 |
| *Hevansia websteri* | BCC36934 | MH532869.1 | Kuephadungphan 2018 |
| *Hevansia websteri* | BCC36541 | MH532868.1 | Kuephadungphan 2018 |
| *Jenniferia cinerea* | BCC36294 | MH532866.1 | Kuephadungphan 2018 |
| *Akanthomyces sulphureus* | BCC36037 | MH532865.1 | Kuephadungphan 2018 |
| *Hevansia novoguineensis* | BCC36270 | MH532864.1 | Kuephadungphan 2018 |
| *Hevansia websteri* | BCC35962 | MH532863.1 | Kuephadungphan 2018 |
| *Akanthomyces kanyawimiae* | BCC34340 | MH532862.1 | Kuephadungphan 2018 |
| *Cordyceps tenuipes* | BCC34337 | MH532861.1 | Kuephadungphan 2018 |
| *Cordyceps tenuipes* | BCC33299 | MH532860.1 | Kuephadungphan 2018 |
| *Cordyceps sp.* | BCC30923 | MH532859.1 | Kuephadungphan 2018 |
| *Akanthomyces kanyawimiae* | BCC30546 | MH532858.1 | Kuephadungphan 2018 |
| *Cordyceps javanica* | BCC29256 | MH532857.1 | Kuephadungphan 2018 |
| *Cordyceps javanica* | BCC29254 | MH532856.1 | Kuephadungphan 2018 |
| *Cordyceps javanica* | BCC28600 | MH532855.1 | Kuephadungphan 2018 |
| *Cordyceps javanica* | BCC28596 | MH532854.1 | Kuephadungphan 2018 |
| *Hevansia novoguineensis* | BCC28811 | MH532853.1 | Kuephadungphan 2018 |
| *Cordyceps javanica* | BCC26304 | MH532851.1 | Kuephadungphan 2018 |
| *Akanthomyces sulphureus* | BCC23822 | MH532850.1 | Kuephadungphan 2018 |
| *Akanthomyces sulphureus* | BCC23821 | MH532849.1 | Kuephadungphan 2018 |
| *Akanthomyces sulphureus* | BCC23820 | MH532848.1 | Kuephadungphan 2018 |
| *Cordyceps cateniannulata* | BCC22822 | MH532847.1 | Kuephadungphan 2018 |
| *Hevansia novoguineensis* | BCC22912 | MH532846.1 | Kuephadungphan 2018 |
| *Hevansia novoguineensis* | BCC22911 | MH532845.1 | Kuephadungphan 2018 |
| *Akanthomyces sp.* | BCC22483 | MH532844.1 | Kuephadungphan 2018 |
| *Akanthomyces sp.* | BCC22600 | MH532843.1 | Kuephadungphan 2018 |
| *Cordyceps javanica* | BCC22477 | MH532842.1 | Kuephadungphan 2018 |
| *Cordyceps tenuipes* | BCC21356 | MH532841.1 | Kuephadungphan 2018 |
| *Akanthomyces kanyawimiae* | BCC21288 | MH532840.1 | Kuephadungphan 2018 |
| *Akanthomyces sulphureus* | BCC20128 | MH532839.1 | Kuephadungphan 2018 |
| *Cordyceps tenuipes* | BCC19941 | MH532838.1 | Kuephadungphan 2018 |
| *Akanthomyces sp.* | BCC19923 | MH532837.1 | Kuephadungphan 2018 |
| *Akanthomyces sp.* | BCC19921 | MH532836.1 | Kuephadungphan 2018 |
| *Akanthomyces sp.* | BCC19511 | MH532835.1 | Kuephadungphan 2018 |
| *Cordyceps fumosorosea* | BCC20180 | MH532834.1 | Kuephadungphan 2018 |
| *Cordyceps cateniobliqua* | BCC18661 | MH532833.1 | Kuephadungphan 2018 |
| *Hevansia novoguineensis* | BCC47879 | MH532832.1 | Kuephadungphan 2018 |
| *Hevansia novoguineensis* | CBS 610.80 | MH532831.1 | Kuephadungphan 2018 |
| *Gibellula trimorpha* | BCC 36526 | NR_182401.1 | Kuephadungphan et. al 2022 |
| *Gibellula solita* | BCC 45574 | NR_182400.1 | Kuephadungphan et. al 2022 |
| *Gibellula parvula* | BCC 48888 | NR_182399.1 | Kuephadungphan et. al 2022 |
| *Gibellula longicaudata* | BCC 40861 | NR_182398.1 | Kuephadungphan et. al 2022 |
| *Gibellula shennongjiaensis* | GZAC SNJ2012 | NR_158478.1 | Zou et. al 2015 |
| *Gibellula queenslandica* | BRIP 72767a | NR_189995.1 | Tan and Shivas 2023 |
| *Gibellula sp.* | BCMU GP01 | AB237661.1 | Yokoyama et. al 2005 |
| *Gibellula formosana* |  | AB100360.1 | Yokoyama and Hara 2003 |
| *Hevansia novoguineensis* | CBS 610.80 | NR_169678.1 | Kuephadungphan 2020 |
| *Gibellula floridensis* | UFSI_5 | PP915747 | **This study** |
| *Metarrhizium anisopliae* | ARSEF 7487 | NR_132017.1 | Schneider et. al 2015 |

| **Supplemental Table S3.** Comparative measurements and morphological characteristics for *G. floridensis*, *G. leiopus*, and *G. pulchra* | | | | | | | | | | | | |
| --- | --- | --- | --- | --- | --- | --- | --- | --- | --- | --- | --- | --- |
| Species | Mycelium color | Synnemata | Conidiophore length µm | Conidiophore width µm | Conidial head diameter µm | Vesicle | Metulae length µm | Metulae width µm | Phialide length µm | Phialide width µm | Conidia length µm | Conidia width µm |
| *G. floridensis* | Yellow, fading to tan with age | Present, numerous, clavate tips | 64-100 | 7.0-16.0 | 41-56 | Present | 8.0-14.0 | 3.0-7.0 | 8.0-11.0 | 2.0-4.0 | 5.0-7.0 | 1.5-3.0 |
| *G. leiopus* | white/yellowish white/yellow/grayish/cream-yellow | Present, numerous, cylindrical/slightly clavate/conical/rounded | 24-80 | 4.4-11.1 | 24-56 | Present | 7.5-10.7 | 3.7-6.4 | 7.9-11.5 | 2.4-3.4 | 3-5.4 | 1.6-2.5 |
| *G. pulchra* | white/yellowish white/pale yellow/pastel yellow/grayish yellow/orange white/pale orange/light orange/yellowish | Present, solitary/numerous, cylindric/slightly enlarged above | 110-640 | 7.9-10.3 | 30-48 | Present | 7.9-9.9 | 5.2-6.4 | 6.4-10.3 | 2-2.4 | 3.5-6 | 1.5-2.5 |

| **Supplemental Table S4.** Overview of raw sequencing data | | | | | |
| --- | --- | --- | --- | --- | --- |
| Sample information | Seq number | Base number (bp) | Mean length (bp) | Min length (bp) | Max length (bp) |
| LA_R1 | 40170 | 11801623 | 293.792 | 50 | 592 |
| LA_R2 | 25864 | 7716310 | 298.3417 | 51 | 592 |
| LA_R3 | 44982 | 13213380 | 293.7482 | 50 | 592 |
| LB1_R1 | 38428 | 11971004 | 311.5177 | 51 | 586 |
| LB1_R2 | 41275 | 12543121 | 303.8915 | 50 | 592 |
| LB1_R3 | 49086 | 14993032 | 305.4442 | 50 | 592 |
| LB2_R1 | 47126 | 13728283 | 291.3102 | 50 | 581 |
| LB2_R2 | 46147 | 14323893 | 310.3971 | 50 | 592 |
| LB2_R3 | 52844 | 16483332 | 311.9244 | 50 | 584 |
| LB3_R1 | 42806 | 12561095 | 293.4424 | 50 | 592 |
| LB3_R2 | 41629 | 12294116 | 295.3258 | 50 | 592 |
| LB3_R3 | 34142 | 9934656 | 290.9805 | 50 | 592 |
| LB4_R1 | 35813 | 11033981 | 308.0999 | 51 | 592 |
| LB4_R2 | 42727 | 12554793 | 293.8375 | 50 | 591 |
| LB4_R3 | 39148 | 11487832 | 293.4462 | 50 | 587 |
| LAT_R1 | 57070 | 17026425 | 298.3428 | 52 | 590 |
| LAT_R2 | 41093 | 12311320 | 299.5965 | 51 | 587 |
| LAPL_R1 | 47130 | 13486941 | 286.1647 | 50 | 590 |
| LAPL_R2 | 41349 | 12037038 | 291.1083 | 52 | 589 |
| LAPL_R3 | 43593 | 12500368 | 286.7517 | 50 | 581 |
| LALV_R1 | 34709 | 9280570 | 267.3822 | 52 | 579 |
| LALV_R2 | 44980 | 12489674 | 277.6717 | 51 | 590 |
| LB1PL_R1 | 37876 | 10824966 | 285.8001 | 50 | 590 |
| LB1PL_R2 | 39338 | 11132948 | 283.0075 | 50 | 592 |
| LB1PL_R3 | 38743 | 11242376 | 290.1783 | 50 | 592 |
| LB1LV | 31115 | 8641374 | 277.7237 | 54 | 591 |
| LB2LV | 31821 | 8248576 | 259.218 | 51 | 591 |
| LB3LV | 30236 | 8431929 | 278.8705 | 54 | 573 |
| LB4LV | 31122 | 8790478 | 282.4522 | 50 | 591 |
| LB1T | 43851 | 12844726 | 292.9175 | 50 | 591 |
| LB2T | 28447 | 8520846 | 299.5341 | 57 | 592 |
| LB3T | 44527 | 13007583 | 292.128 | 60 | 592 |
| LB4T | 39005 | 11641246 | 298.4552 | 50 | 590 |
| LB2PL_R1 | 27041 | 7343432 | 271.5666 | 50 | 592 |
| LB2PL_R2 | 31763 | 9121391 | 287.1703 | 50 | 591 |
| LB2PL_R3 | 34523 | 9326838 | 270.163 | 53 | 592 |
| LB3PL_R1 | 30264 | 8243104 | 272.3732 | 54 | 579 |
| LB3PL_R2 | 35649 | 10396491 | 291.6349 | 51 | 588 |
| LB3PL_R3 | 77923 | 22657975 | 290.7739 | 51 | 592 |
| LB4PL_R1 | 45775 | 13348936 | 291.6207 | 51 | 590 |
| LB4PL_R2 | 36444 | 10686574 | 293.2327 | 52 | 592 |
| LB4PL_R3 | 34219 | 10107383 | 295.3734 | 55 | 590 |
